# Succinyl-proteome profiling of a high taxol containing hybrid *Taxus* species (*Taxus* × *media*) revealed involvement of succinylation in multiple metabolic pathways

**DOI:** 10.1038/srep21764

**Published:** 2016-02-23

**Authors:** Chenjia Shen, Jie Xue, Tao Sun, Hong Guo, Lei Zhang, Yijun Meng, Huizhong Wang

**Affiliations:** 1 College of Life and Environmental Sciences, Hangzhou Normal University, Hangzhou 310036, China; 2 Department of Plant Pathology, North Carolina State University, Raleigh, North Carolina 27607.

## Abstract

Protein lysine succinylation, a ubiquitous protein post-translational modification among eukaryotic and prokaryotic cells, represents a vital regulator of various metabolic processes. However, little is known about its functions and cellular distribution in *Taxus* × *media*, which is a hybrid *Taxus* species containing a high content of taxol. In this study, LC-MS/MS was used to identify peptides enriched by immune-purification with high-efficiency succinyl-lysine antibody. A total of 193 succinylated proteins and 325 succinylation sites were identified. The bioinformatics analysis indicated that these succinylated proteins were involved in a wide range of cellular functions from metabolism to protein binding and showed diverse subcellular localizations. Furthermore, our findings suggested that lysine succinylation in *Taxus* × *media* involved a diverse array of metabolic processes and protein–protein interactions. Many enzymes involved in multiple metabolic pathways, such as glycolysis, pyruvate metabolism, the tricarboxylic acid cycle and carbon fixation, were identified as substrates for lysine succinylation, suggesting the presence of a common mechanism underlying the participation of succinylation in metabolic regulation. These results provide the first comprehensive view of the succinylome of *Taxus* × *media* and may catalyze future biological investigation of succinylation.

The physicochemical properties, space conformation and stability of proteins are reportedly influenced by protein post-translational modifications (PTMs), a dynamic and reversible way for protein chemical modifications at the post-translational level^1^,^2^. PTMs increase the functional diversity of proteins by adding covalent modifications, including phosphorylation, ubiquitination, glycosylation, methylation and acetylation^3^,^4^,^5^,^6^,^7^,^8^. Since the first identification and verification in prokaryote (*Escherichia coli*) proteins, lysine succinylation (Ksuc) has been identified in many proteins in different species, such as *Saccharomyces cerevisiae*, *Drosophila melanogaster* and *Mus mucsculus*^9^. Identifying and analyzing lysine succinylation is an efficient approach to gain a comprehensive understanding of the diversified functions of protein in dramatic structural and enzymatic activity changes in metabolism^9^,^10^.

The natural product Taxol® (generic name paclitaxel) was first isolated from the bark of *Taxus brevifolia* decades ago^11^. As a highly effective antitumor agent, taxol has been well documented and approved by the US Food and Drug Administration for the treatments of various cancers since the 1990 s^12^. There have been many studies on the importance of, and the urgent need for, taxol in both clinical and scientific research. Recently, the biosynthetic pathway of taxol was investigated in *Taxus* × *media*, a hybrid *Taxus* species containing a high content of taxol^13^,^14^. A large number of genes have been cloned and analyzed to reveal the biosynthetic pathway leading to this complex diterpenoid compound^15^,^16^,^17^,^18^. However, PTMs in the metabolism-related enzymes in *Taxus* × *media* is largely unknown. Interestingly, most of the previously identified succinylated proteins are enzymes involved in multiple metabolic pathways and proteins important for the regulation of central metabolisms^19^,^20^. Systematic identification of the lysine succinylome of *Taxus* × *media* may facilitate us for further understanding of the biosynthetic pathway of taxol and the molecular basis for the higher taxol content in *Taxus* × *media*.

In the present work, we identified 325 succinylation sites on 193 proteins with remarkably diverse biological functions and cellular localizations in *Taxus* × *media*. Moreover, three unique motifs were found through bioinformatics analysis of the sequences flanking the succinylation sites. To our knowledge, these results provide the first comprehensive view of the succinylome of *Taxus* × *media* and will catalyze future biological investigation of lysine succinylation.

## Results

### Proteome-wide analysis of lysine succinylation sites on the proteins in *Taxus* × *media*

Lysine succinylation is an evolutionarily conserved, but highly dynamic phenomenon under various cellular conditions in both prokaryotic and eukaryotic cells^7^,^9^,^10^. To date, the succinylome in *Taxus* × *media* has not yet been reported. In our study, we combined lysine-succinylated peptides enrichment with highly sensitive mass spectrometry (MS) and bioinformatics tools to reveal the systematic lysine-succinylated sites and proteins in this species.

**Figure 1:**
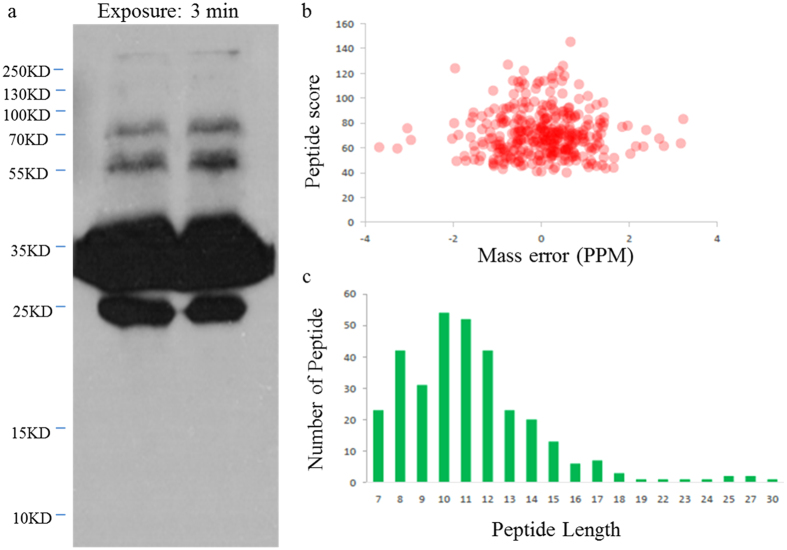
QC validation of LC-MS/MS data. (**a**) Western blotting analysis of 5102WB with pan anti-succinylation antibody demonstrates the presence of succinylated proteins. In total, 40 μg of whole tissue lysate was loaded in one lane, and primary antibody was diluted by 1:1000. (**b**) The distribution of mass error. (**c**) T-distribution of succinylated peptides based on their length.

Western-blot analysis by an antibody recognizing succinyl-lysine was performed to characterize the extent of lysine succinylation on the proteins of *Taxus* × *media*. As expected, multiple protein bands spanning a wide mass-range were detected, indicating the presence of diverse succinylated proteins (Fig. 1a). In this study, as no significant signal of succinylation on histones was found in western blot experiment, we were not focused in histone marks. Therefore, the current workflow is not suitable for the identification of histone modifications. Because of the current workflow and the low-abundant of succinylation in histones, only one histone succinylation site was identified in this work (Supplementary Table S1). Liquid chromatograph-mass spectrometer (LC-MS/MS) analysis identified 325 succinylation sites on 193 proteins in *Taxus* × *media* with high confidence, and these peptides exhibited distinct abundance depending on their lengths (Supplementary Table S2). First, the MS data validation was performed by checking the mass error of all identified peptides. The distribution of mass errors was near zero and most values were <0.02 Da, indicating that the mass accuracy of the MS data met the requirement (Fig. 1b). Second, the lengths of most peptides were distributed within 8 to 20 amino-acid residues, which was in agreement with the property of tryptic peptides, and thus sample preparation met the technically standard (Fig. 1c).

Of all the identified succinylated proteins, ~65% (126/193) possessed a single succinylated site, 17.6% (34/193) possessed two and 2.6% (5/193) possessed three sites. The average degree of succinylation was 1.68 (325/193). Notably, eight proteins had five or more succinylated sites. Three proteins, including a pyridine nucleotide-disulfide oxidoreductase, an ATPase and a nucleoside phosphorylase had the most extensively succinylated sites (more than seven independent lysine residues) (Supplementary Table S3).

### Characterization of lysine succinylome of *Taxus* × *media*

**Figure 2:**
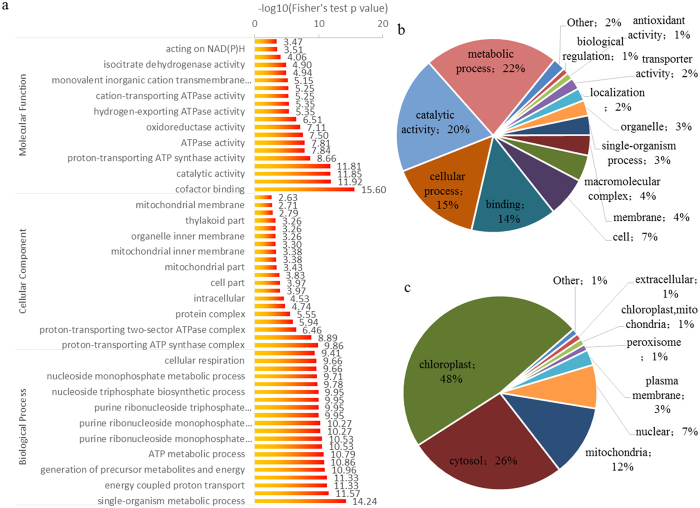
Characterization of lysine succinylome of *Taxus* × m*edia*. (**a**) Enrichment analysis of succinylated proteins based on the classification of GO annotation in terms of molecular function, cellular component and biological process. Proportion of the succinylated proteins in different biological functions (**b**) and subcellular locations (**c**).

To better characterize the succinylated proteins in *Taxus* × *media*, we searched the UniProt-GOA database (www.http://www.ebi.ac.uk/GOA/) to annotate all these proteins with their Gene Ontology (GO) functional classification. Our data showed that succinylation occurred on proteins categorized as participating in diverse biological processes, cellular components and molecular functions. Among these GO terms, the largest classes of succinylated proteins were ‘metabolic process’ in biological process and ‘catalytic activity’ associated with molecular function (Fig. 2a). In detail, 22% of all the annotated succinylated proteins were involved in metabolic processes, and 20% were catalytic enzymes. Moreover, another large succinyl protein group associated with biological function was ‘cellular process’, representing 15% of all annotated succinylated proteins (Fig. 2b).

Few studies on the subcellular localizations of the succinyl-proteins have been reported. Therefore, the subcellular distribution of the succinylated proteins was also analyzed and classified in *Taxus* × *media* (Fig. 2c). In total, 48% (92/193) of the succinylated proteins are located in the chloroplasts, 23% (51/193) in the cytosol, 12% (23/193) in the mitochondria and 7% (14/193) in the nucleus.

### Motifs analysis for identified lysine-succinylated peptides

**Figure 3:**
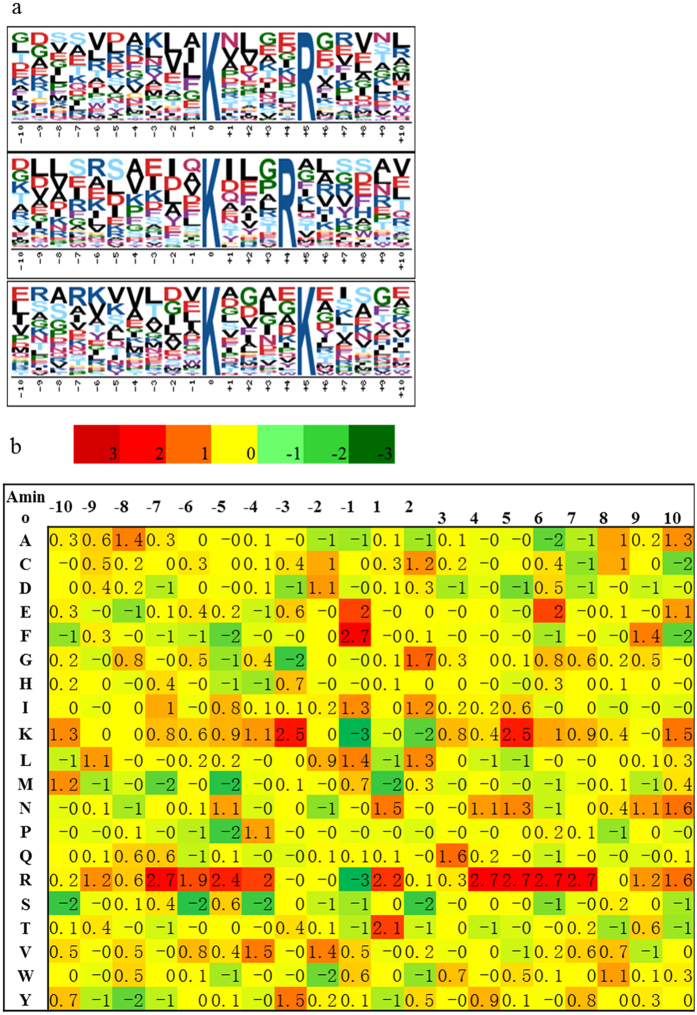
Bioinformational analysis of lysine succinylation sites. (**a**) Plot shows relative abundance of amino acids flanking succinylated lysine; the relative abundance was calculated and then schematically represented by an intensity map. The intensity map shows enrichment of amino acids in specific positions of succinylated lysine (10 amino acids upstream and downstream of the succinylation site). (**b**) Probability sequence motifs of succinylation sites consisting of 10 residues surrounding the targeted lysine residue using Motif-X. Three significantly enriched succinylation site motifs were identified.

Motif analysis is conducive to evaluating the features of the succinylated proteins in *Taxus* × *media*, thus the Motif-X program was used to search for the sequence motifs in all identified succinylated peptides. Three preferred sequence patterns: **K^suc^****R** (Motif I), **K^suc^***R** (Motif II) and **K^suc^****K^suc^** (Motif III) (* indicates a random amino acid residue and K^suc^ indicates succinylated-K) were identified as conserved succinylation site motifs (Fig. 3a). The sequence logos showed a strong bias for an arginine (R) downstream of the succinylated lysines. For Motif I, an R was observed on the fourth position after the K^suc^; while another R occurred most frequently downstream of K^suc^ in Motif II (R was observed on the third position after K^suc^) (Table 1).

Furthermore, a logo reflecting relative frequency of amino acids in specific positions of succinyl-21-mers (10 amino acids upstream and downstream of the modification site) compared with that of nonsuccinyl-21-mers (10 amino acids upstream and downstream of the non-modification site) was constructed to reflect whether there was a significant frequency of specific amino acids flanking the succinylated lysine site. The data showed that K had the lowest frequency in positions −1 and +2 and the highest frequency in positions −3 and +5 in the motifs (Fig. 3b).

### Many succinylated proteins are engaged in metabolic pathways

Pathway analysis of our succinylome data showed that many succinylated proteins were involved in multiple metabolic pathways, such as glycolysis, pyruvate metabolism, the tricarboxylic acid (TCA) cycle and carbon fixation.

**Figure 4:**
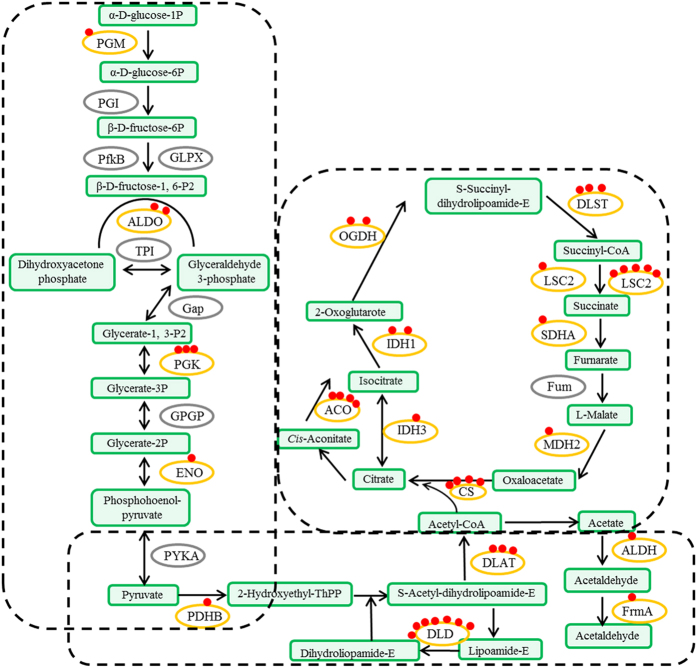
Succinylated enzymes involved in major metabolic pathways (glycolysis, pyruvate metabolism and the TCA cycle). PGM: α-D-phosphohexomutase; ALDO: fructose-bisphosphate aldolase; PGK: phosphoglycerate kinase; ENO: enolase; PDHB: pyruvate dehydrogenase; DLD: dihydrolipoamide dehydrogenase; DLAT: pyruvate dehydrogenase; ALDH: aldehyde dehydrogenase; frmA: S-(hydroxymethyl) glutathione dehydrogenase; CS: citrate synthase; ACO: aconitate hydratase; IDH1: isocitrate dehydrogenase; IDH3: isocitrate dehydrogenase (NAD+); OGDH: 2-oxoglutarate dehydrogenase; DLST: dihydrolipoamide succinyltransferase; LSC1: succinyl-CoA synthetase 1; LSC2: succinyl-CoA synthetase 2; SDHA: succinate dehydrogenase; and MDH2: malate dehydrogenase.

Glycolytic enzymes involved in the conversion of glucose to pyruvate, which is one of the major products of glycolysis, were reported to be substrates of lysine succinylation. In total, four out of 11 glycolytic enzymes were identified as succinylated proteins, including α-D-phosphohexomutase (PGM), fructose-bisphosphate aldolase (ALDO), phosphoglycerate kinase (PGK) and enolase (ENO). Most of these are also succinylated in *E. coli*, *Mycobacterium tuberculosis* and mammals, indicating potential conserved function of PTM in the regulation of glycolytic metabolism^21^. Furthermore, conversion of pyruvate to acetyl-CoA and carbon dioxide (CO_2_) is catalyzed by the pyruvate dehydrogenase complex^22^. In this complex, five components were found to be succinylated at lysine: pyruvate dehydrogenase (PDHB), dihydrolipoamide dehydrogenase (DLD), pyruvate dehydrogenase (DLAT), aldehyde dehydrogenase (ALDH) and S-(hydroxymethyl) glutathione dehydrogenase (frmA). In particular, DLD contained the largest number of succinylated sites (seven sites). In the TCA cycle, ten key enzymes were lysine-succinylated: citrate synthase (CS), aconitate hydratase (ACO), isocitrate dehydrogenase (IDH1), isocitrate dehydrogenase (NAD+) (IDH3), oxoglutarate dehydrogenase (OGDH), dihydrolipoyllysine-residue succinyltransferase (DLST), succinyl-CoA synthetases (LSC1 and LSC2), succinate dehydrogenase (SDHA) and malate dehydrogenase (MDH2) (Fig. 4 and Supplementary Table S4). Three representative LC-MS/MS spectra of succinyl-peptides from the TCA cycle, IDH1, LSC1 and LSC2, are shown in Supplementary Fig. S1–S3.

**Figure 5:**
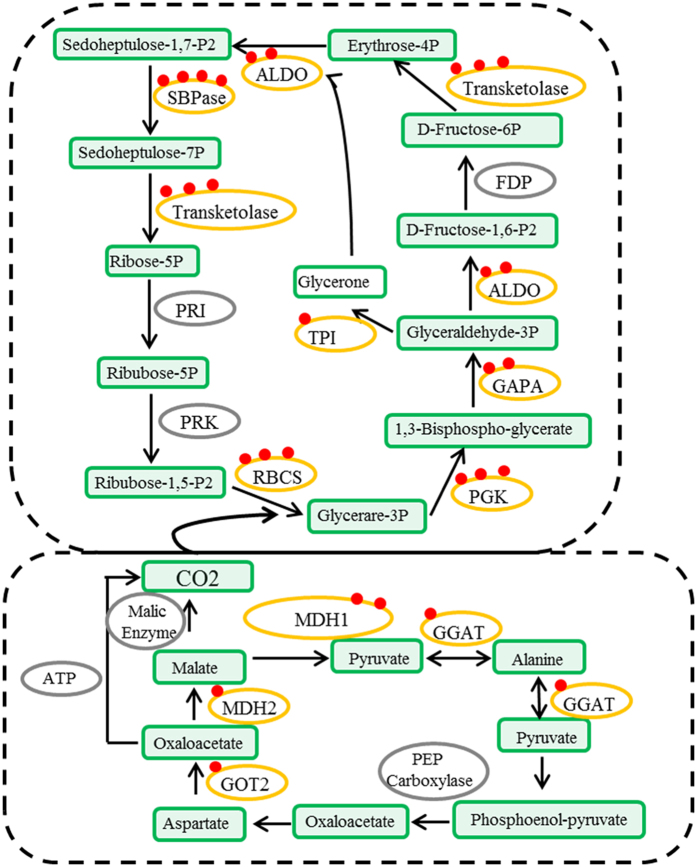
Succinylated enzymes involved in major metabolic pathways (carbon fixation). ALDO: fructose-bisphosphate aldolase; SBPase: sedoheptulose bisphosphatase; RBCS: ribulose bisphosphate carboxylase; PGK: phosphoglycerate kinase; GAPA: glyceraldehyde-3-phosphate dehydrogenase; TPI: triosephosphate isomerase; GOT2: aspartate aminotransferase; MDH1: malate dehydrogenase1; MDH2: malate dehydrogenase2; and GGAT: glutamate–glyoxylate aminotransferase.

Carbon fixation is an important process in plant metabolism^23^. In total, 11 enzymes related to carbon fixation were identified as succinylated proteins, including ALDO, sedoheptulose bisphosphatase (SBPase), transketolase, ribulose bisphosphate carboxylase (RBCS), PGK, glyceraldehyde-3-phosphate dehydrogenase (GAPA), triosephosphate isomerase (TPI), aspartate aminotransferase (GOT2), malate dehydrogenase (MDH1), MDH2 and glutamate-glyoxylate aminotransferase (GGAT) (Fig. 5 and Supplementary Table S5).

### Protein–protein interactions (PPIs) network of *Taxus* × *media* lysine succinylation substrates suggest ubiquitous involvement of succinylated proteins

**Figure 6:**
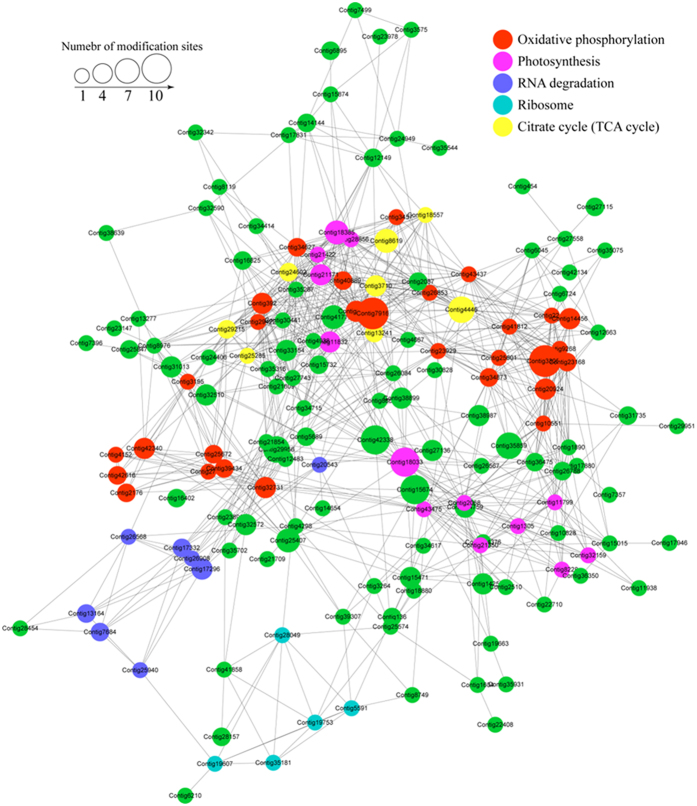
Interaction network of lysine-succinylated proteins analyzed by Cytoscape software (version 3.0.1). The lysine-succinylated proteins in the top five clusters are shown in red, light purple, blue, cyan and yellow, respectively. Red indicates cluster ‘Oxidative phosphorylation’, light purple indicates cluster ‘Photosynthesis’, blue indicates cluster ‘RNA degradation’, cyan indicates cluster ‘Ribosome’, yellow indicates cluster ‘Citrate cycle’ and green indicates other lysine-succinylated proteins in *Taxus* × *media*.

To further investigate the biological processes regulated by succinylation, we analyzed the PPIs among the identified 193 succinylated proteins. The *Taxus* × *media* PPI network had 162 succinylated proteins as nodes, connected by a large number of identified direct physical interactions obtained from the STRING database (Supplementary Table S6). Furthermore, we constructed a high quality image as an overview of the PPIs of succinylated proteins in *Taxus* × *media* (Fig. 6).

The top five enriched interaction clusters from the data analysis are indicated by different colors in Fig. 6. Interestingly, the first cluster (cluster 1) consisted of 29 oxidative phosphorylation proteins, which had an average degree of nodes of 16.8 (Supplementary Fig. S4 and Table S7). The second cluster (cluster 2) consisted of 13 photosynthesis-associated proteins, which had an average degree of nodes of 18.5 (Supplementary Fig. S5 and Table S8). Eight RNA degradation-related proteins were classed into cluster 3, which had an average degree of nodes of 7.3 (Supplementary Fig. S6 and Table S9). Five ribosome-associated proteins were identified in cluster 4, with an average degree of nodes of 6.6 (Supplementary Fig. S7 and Table S10). The last enriched interaction cluster (cluster 5) consisted of eight TCA cycle-related proteins, with an average degree of nodes of 15.8 (Supplementary Fig. S8 and Table S11).

## Discussion

PTM is a chemical modification that is dynamic, evolutionarily conserved and sometimes reversible. As a typical PTM, acetylation has been well-studied in recent years^24^. Acetylation can provide an elegant way to coordinate extracellular signaling and intracellular metabolism by utilizing metabolic enzymes^25^. Beyond protein acetylation, lysine succinylation was also reported to be another kind of PTMs by activating a reaction intermediate during the transfer of a succinyl group from succinyl-CoA to homoserine^26^. Using MS as a powerful tool, as well as antibody-based affinity enrichment of succinylated lysine residues, we analyzed and validated 325 lysine succinylation sites in *Taxus* × *media*.

In our study, we compared the *Taxus* × *media* succinylome with previously reported succinylomes of *M. tuberculosis*, *E. coli* and *Toxoplasma gondii*^20^,^21^,^27^. There were fewer succinylated proteins and succinylated sites in *Taxus* × *media* than in *M. tuberculosis*, *S. cerevisiae* and *E. coli* (Supplementary Table S12). The lack of an available genome database and incomplete transcriptome data may be the major causes of the smaller number of succinylated proteins identified in *Taxus* × *media* in the present study^28^. The average number of succinylated sites per protein in *Taxus* × *media* was much smaller than of all other species, suggesting that many succinylation sites remained unidentified in *Taxus* × *media*^19^. In recent years, several mammalian succinylomes revealed that lysine succinylation has positive effects on the activities of enzymes involved in mitochondrial metabolism^29^,^30^. Systematic profiling of the mouse succinylome showed that 60% of mitochondrial proteins were succinylated in embryonic fibroblasts, compared to 23% in liver tissue^30^. In *Toxoplasma gondii*, the largest group of succinylated proteins is also located in mitochondria (26%)^27^; however, only 12% of the succinylated proteins are located in mitochondria in *Taxus* × *media* (Fig. 2c). It is noteworthy that the subcellular localizations of the succinylated proteins are different in various eukaryotic organisms. In contrast, nearly half of the succinylated proteins (48%) showed chloroplast location and one-quarter of the succinylated proteins (26%) were cytosol-located proteins in *Taxus* × *media* (Fig. 2c). It is likely that MS analysis more readily detected the more abundant proteins, such as chloroplast proteins, which accounted for the larger proportion in plants.

Emerging evidence suggests that succinylation is essential for regulating cellular metabolism at different levels^21^,^24^,^30^. Enzymes are ubiquitous proteins that catalyze a large number of chemical reactions in both prokaryotes and eukaryotes^31^. Recently, proteomic studies on succinylation indicated that several metabolic enzymes are over-represented within the succinylomes^19^,^30^,^32^. Our data are consistent with previous reports concerning various prokaryotes and eukaryotes, suggesting the presence of a common mechanism behind participation of succinylation in metabolic regulation^19^,^21^,^27^. Pathway analysis showed that 138 succinylated proteins were involved in multiple metabolic pathways. Our study focused on the metabolic pathways, including glycolysis, the TCA cycle, and carbohydrate and pyruvate metabolism.

In microbes and mammals, most glycolytic enzymes are succinylated^19^,^20^,^21^. In *Taxus* × *media*, succinylation occurred on four key enzymes, suggesting a potential conserved function of succinylation in the regulation of glycolytic flux. IDH1 catalyzes the rate-limiting step of the TCA cycle – the conversion of isocitrate to 2-oxoglutarote and CO_2_^33^. The importance of the succinylated lysine residues of isocitrate dehydrogenase (K100 and K242) in its activity was revealed in *E. coli* by mutagenesis analysis^9^. In our study, we analyzed two Ksucc sites (K117 and K155) on IDH1 that were critical for catalysis (Supplementary Fig. S1). It is in agreement with previous reports in other species^9^,^30^. Succinyl-CoA, a cofactor of enzyme-mediated lysine succinylation, is a critical metabolic intermediate in the TCA cycle. Succinyl-CoA ligase (LSC) catalyzes the nucleotide-dependent conversion of succinyl-CoA to succinate^34^. LSC1 contains five lysine succinylation sites and LSC2 contains only one lysine succinylation site in *Taxus* × *media* (Supplementary Figs S2 and S3). Our data indicated a potential function in turnover of succinyl-CoA production in the regulation of enzymatic activity by lysine succinylation^21^.

PPIs are essential to numerous biological processes^35^. For example, extracellular signals from the exterior of a cell are mediated into the cell by PPIs between different signaling molecules^36^. In previous studies, the global PPI network of succinylated proteins was only determined in some prokaryotic species^20^,^21^. Our study provided the first high-quality interaction network of the succinylated proteins in plants. Interestingly, the sub network of the TCA cycle showed relatively high enrichment in *Taxus* × *media* (Supplementary Fig. S5). Eight proteins were found to be grouped into cluster 5 (TCA cycle-related proteins). These results confirmed an important regulatory role of succinylation modification in the TCA cycle. The degree of each node is an important index to evaluate the strength of protein connection in a network^21^. Our data speculated that the proteins classed into clusters 1, 2 and 5 showed high degrees, suggesting that these proteins showed higher connectivity than the proteins of clusters 3 and 4.

We presented the first large-scale succinyl-proteome of *Taxus* × *media*, an important medicinal plant. We analyzed 325 lysine succinylation sites occurring on 193 proteins in *Taxus* × *media*. These succinylated proteins may be involved in a broad spectrum of functions ranging from catalytic activity to protein binding, and were distributed in various cellular compartments, speculating that protein succinylation was likely to be vital in regulation of the physiology and biochemical processes in *Taxus* × *media*. Moreover, our comprehensive succinyl-proteome analysis of *Taxus* × *media* may provide a basic resource for in-depth functional examination of these succinylated proteins in dramatic structural and enzymatic activity changes in metabolism. It may help us to further understand the biosynthetic pathways and the molecular basis for higher Taxol content in *Taxus* × *media*.

## Methods

### Plant sample and lysate preparation

The bark of two-year-old hybrid Taxus species, *Taxus* × *media*, trees was used for protein extraction. The bark from the hybrid was first dipped in liquid nitrogen and sonicated three times on ice using a high intensity ultrasonic processor (type number JY92-IIN, Scientz, Ningbo, China) in lysis buffer. The exact parameters are: power 195 W, sonic disruption over intervals up to 5 min (ultrasound 3 sec, stop 3 sec, alternately). The content of lysis buffer is: 8 M urea, 1% Triton-100, 10 mM DTT and 0.1% Protease Inhibitor Cocktail IV, 3 μM TSA, 50 mM NAM, 2 mM EDTA. Then, the debris was removed by centrifugation at 20,000 g at 4 °C for 15 min. Finally, the protein sample was precipitated with cold 15% TCA for 2 h at −20 °C. The supernatant was discarded by centrifugation at 20,000 g at 4 °C for 10 min, the supernatant was discarded. The remaining precipitate was washed with pre-cooling acetone for three times. The protein was redissolved in buffer (8 M urea, 100 mM NH_4_CO_3_, pH 8.0). A 2-D Quant kit (GE Healthcare, Uppsala, Sweden) was used to determine the protein concentration according to the manufacturer’s instructions.

### Trypsin digestion

For digestion, the protein solution was reduced with DTT (10 mM) for 1 h at 37 °C and alkylated with iodoacetamide (20 mM) for 45 min at room temperature in darkness. For trypsin digestion, the protein sample was diluted in 100 mM NH_4_HCO_3_. Finally, trypsin (Promega (Beijing) Biotech Co., Ltd) was added at 1:50 trypsin-to-protein ratio for the first digestion overnight. To ensure protein digested completely, additional 1:100 trypsin-to-protein ratio for a second 4 h-digestion. The digested peptides were lyophilized in a SpeedVac (Thermo Scientific) and stored at −80 °C.

### HPLC Fractionation and Affinity Enrichment

The protein sample was fractionated into factions by reverse-phase HPLC (high pH) using Agilent 300Extend C18 column (5 μm particles, 4.6 mm ID, 250 mm length). Briefly, peptides were first separated with a gradient of 2% to 60% acetonitrile in ammonium bicarbonate (10 mM, pH 10) over 80 min into 80 fractions. Then, the peptides were combined into 8 fractions and dried by vacuum centrifuging.

To enrich Ksu peptides, a NETN buffer (100 mM NaCl, 1 mM EDTA, 50 mM Tris-HCl, 0.5% NP-40, pH 8.0) was used to dissolve the tryptic peptides. Then, the tryptic peptides were incubated with pre-washed antibody beads (PTM Biolabs) at 4 °C overnight with gentle shaking. The beads were washed four times with NETN buffer and twice with ddH_2_O. The bound peptides were eluted from the beads with 0.1% trifluoroacetic acid (TFA). The eluted fractions were combined and vacuum-dried. The resulting peptides were cleaned with C_18_ ZipTips (Millipore) according to the manufacturer’s instructions, followed by LC-MS/MS analysis.

### Western Blotting

Western blotting assays were performed using protein lysates from tachyzoites by 12% SDS-PAGE. After they were transferred to the nitrocellulose membrane (Millipore), the membranes were incubated in blocking buffer (0.05% Tween 20 and 5% nonfat milk powder in PBS). Succinylated lysines were detected using rabbit-derived polyclonal antisuccinyl lysine antibodies (PTM Biolabs, Hangzhou, China) diluted in blocking buffer at 1:1000 overnight at 4 °C. Membranes were washed and incubated with horseradish peroxidase-conjugated antirabbit secondary antibody (Sigma) diluted at 1:2000 and chemiluminescence substrate for detection (Sigma).

### Proteomic Analysis by LC-MS/MS

Peptides were dissolved in 0.1% formic acid (FA), loaded on a reversed-phase pre-column (Acclaim PepMap 100, Thermo Scientific). A reversed-phase analytical column (Acclaim PepMap RSLC, Thermo Scientific) was used for peptide separation. The gradient solvent B (0.1% FA in 98% ACN) was comprised of an increase from 7% to 20% for 20 min, 20% to 35% for 8 min and climbing to 80% within 2 min then holding at 80% for the last 5 min, all at a constant flow rate of 300 nl/min on an EASY-nLC 1000 UPLC system, the resulting peptides were analyzed by Q ExactiveTM Plus hybrid quadrupole-Orbitrap mass spectrometer (ThermoFisher Scientific).

The peptides were subjected to NanoSpray Ionization source followed by tandem mass spectrometry (MS/MS) in Q ExactiveTM Plus (Thermo Scientific) coupled online to the UPLC. Intact peptides were detected in the Orbitrap at a resolution of 70,000. Peptides were selected for MS/MS using NCE setting as 33; ion fragments were detected in the Orbitrap at a resolution of 17,500. A data-dependent procedure that alternated between one MS scan followed by 16 MS/MS scans was applied for the top 16 precursor ions above a threshold ion count of 1.5E4 in the MS survey scan with 10.0 s dynamic exclusion. The electrospray voltage applied was 2.0 kV. Automatic gain control (AGC) was used to prevent overfilling of the ion trap; 5E4 ions were accumulated for generation of MS/MS spectra. For MS scans, the m/z scan range was 350 to 1800 Da. Fixed first mass was set as 100 m/z.0.

### Database Search

The identification of protein and succinylation was processed using MaxQuant with integrated Andromeda search engine (v.1.4.1.2). In our experiment, tandem mass spectra were searched against the transcriptome data, which was downloaded from the published database (NCBI Sequence Read Archive database under the accession numbers SRX156706 and SRX156707) concatenated with reverse decoy database. Based on the published transcriptome data, 40,348 unigenes sequences were obtained by further assembly from the two datasets. Trypsin/P was specified as cleavage enzyme allowing up to 4 missing cleavages, 5 modifications per peptide and 5 charges. Mass error was set to 10 ppm for precursor ions and 0.02 Da for fragment ions. Carbamidomethylation on Cys was specified as fixed modification and oxidation on Met, acetylation on lysine and acetylation on protein N-terminal were specified as variable modifications. False discovery rate (FDR) thresholds for protein, peptide and modification site were specified at 1%. Minimum peptide length was set at 7. All the other parameters in MaxQuant were set to default values. In detail, the default values was described as follow: (“first search” set as none, “main search ppm” set as 4.5, “min score for modified peptides” set as 40, and “min delta score for modified peptides” set as 17. The site localization probability was set as >0.75. Lysine succinylation sites identified with a localization probability of <0.75 were removed.

### The methods bioinformatics analysis

#### Annotation methods

Gene Ontology (GO) annotation proteome was derived from the UniProt-GOA database (www. http://www.ebi.ac.uk/GOA/). Firstly, Converting identified protein ID to UniProt ID and then mapping to GO IDs by protein ID. If some identified proteins were not annotated by UniProt-GOA database, the InterProScan soft would be used to annotated protein’s GO functional based on protein sequence alignment method. Then proteins were classified by Gene Ontology annotation based on three categories: biological process, cellular component and molecular function.

#### Domain Annotation

Functional description of identified protein domains were annotated by InterProScan (a sequence analysis application) based on protein sequence alignment method. InterPro (http://www.ebi.ac.uk/interpro/) is a database that integrates diverse information about protein families, domains and functional sites, and makes it freely available to the public via Web-based interfaces and services. Central to the database are diagnostic models, known as signatures, against which protein sequences can be searched to determine their potential function. InterPro has utility in the large-scale analysis of whole genomes and meta-genomes, as well as in characterizing individual protein sequences.

#### KEGG Pathway Annotation

KEGG Pathways mainly including: Metabolism, Genetic Information Processing, Environmental Information Processing, Cellular Processes, Rat Diseases, Drug development. Kyoto Encyclopedia of Genes and Genomes (KEGG) database was used to annotate protein pathway. Firstly, using KEGG online service tools KAAS to annotated protein’s KEGG database description. Then mapping the annotation result on the KEGG pathway database using KEGG online service tools KEGG mapper.

#### GO/KEGG Pathway Functional Enrichment Analysis

Basing on GO annotation, proteins were classified into three categories, including biological process, cellular compartment and molecular function. For each category, we used Functional Annotation Tool of DAVID Bioinformatics Resources 6.7 to identify enriched GO against the background of Homo sapiens. A two-tailed Fisher’s exact test was employed to test the enrichment of the protein-containing IPI entries against all IPI proteins. Correction for multiple hypothesis testing was carried out using standard false discovery rate control methods. The GO with a corrected *p*-value < 0.05 is considered significant.

Encyclopedia of Genes and Genomes (KEGG) database was used to identify enriched pathways by Functional Annotation Tool of DAVID against the background of Homo sapiens. A two-tailed Fisher’s exact test was employed to test the enrichment of the protein-containing IPI entries against all IPI proteins. Correction for multiple hypothesis testing was carried out using standard false discovery rate control methods. The pathway with a corrected *p*-value < 0.05 was considered significant. These pathways were classified into hierarchical categories according to the KEGG website.

For each category proteins, InterPro (a resource that provides functional analysis of protein sequences by classifying them into families and predicting the presence of domains and important sites) database was researched using Functional Annotation Tool of DAVID against the background of Homo sapiens. A two-tailed Fisher’s exact test was employed to test the enrichment of the protein-containing IPI entries against all IPI proteins. Correction for multiple hypothesis testing was carried out using standard false discovery rate control methods and domains with a corrected *p*-value < 0.05 were considered significant. For the bioinformatics analysis, such as the GO-base and KEGG-base enrichment, all the sequences in the database were used as the background.

### Motif analysis

The motif-x software was used to analysis the model of identified succinylation sites. Peptide sequence with 10 amino acids upstream and downstream of the identified succinylation site from the protein sequence was used as foreground, while all the identified protein sequences (digested with trypsin) were used as background. Analysis parameters: Modified acid amino “central character” set as ‘K’ (lysine), foreground peptides sequence length “width” set at 21, minimal number of peptide occur in one motif “occurrences” set at 20, motif analysis statistics test significance threshold value set at 0.0000001.

The “motif score” is calculated by taking the sum of the negative log probabilities used to fix each position of the motif. As such, higher motif scores typically correspond to motifs that are more statistically significant as well as more specific (i.e., greater number of fixed positions).

The “foreground matches” and “background matches” statistics indicate the number of peptides containing a given motif in those respective data sets following the removal of all peptides containing previously extracted motifs. Because of this iterative “set reduction” strategy, the “foreground matches” and “background matches” statistics may be less than or equal to the total number of instances of a given motif in the whole data set.

The “foreground size” and “background size” statistics indicate the total number of peptides contained in these data sets. The size of these data sets decreases 23 as motifs are extracted (i.e., down a column) due to the fact that peptides are removed from both the foreground and background data sets following motif extraction. The total number of foreground peptides not falling into any extracted motif class can therefore be calculated as the difference between the “foreground size” and the “foreground matches” of the final motif class (e.g., 163 – 32 = 131 unclassified peptides).

The “fold increase” statistic is an indicator of the enrichment level of the extracted motifs. Specifically, it is calculated as (foreground matches/foreground size)/(background matches/background size).

### Motif logo-based Clustering Analysis

All the succinylation substrates categories obtained after enrichment were collated along with their P values, and then filtered for those categories which were at least enriched in one of the clusters with *p*-value < 0.05. This filtered P value matrix was transformed by the function x = −log10 (*p* value). Finally, these x values were z-transformed for each category. These z scores were then clustered by one-way hierarchical clustering (Euclidean distance, average linkage clustering) in Genesis. Cluster membership was visualized by a heat map using the “heatmap.2” function from the “gplots” R-package.

### Protein-protein Interaction Network

All identified succinylated protein name identifiers were searched against the STRING database version 9.1 for protein-protein interactions. Only interactions between the proteins belonging to the searched data set were selected, thereby excluding external candidates. STRING defines a metric called “confidence score” to define interaction confidence; we fetched all interactions that had a confidence score ≥ 0.7 (high confidence). Interaction network form STRING was visualized in Cytoscape. A graph of the clustering algorithm, molecular complex detection (MCODE) was utilized to analyze densely connected regions. MCODE is part of the plug-in tool kit of the network analysis and visualization software Cytoscape.

## Additional Information

**How to cite this article**: Shen, C. *et al.* Succinyl-proteome profiling of a high taxol containing hybrid *Taxus*species (*Taxus*× *media*) revealed involvement of succinylation in multiple metabolic pathways. *Sci. Rep.* **6**, 21764; doi: 10.1038/srep21764 (2016).

## Supplementary Material

Supplementary Information

## Figures and Tables

**Table 1:** Motifs Analysis for Identified Lysine Succinylated Peptides.

Motif	Motif Score	Foreground		Background	Fold Increase	
		Matches	Size	Matches	Size	
……….K….R…..	5.79	36	318	215	4536	2.39
……….K…R……	3.59	26	282	186	4321	2.14
……….K….K…..	3.4	34	256	296	4135	1.86
