# Supplementary material for: Succinyl-proteome profiling of a high taxol containing hybrid Taxus species (Taxus × media) revealed involvement of succinylation in multiple metabolic pathways
Source: Sci Rep. 2016 Feb 23;6:21764. doi: 10.1038/srep21764 (PMC4763222; doi:10.1038/srep21764)
Supplement: Supplementary Information [file srep21764-s1.pdf]

Succinyl-proteome profiling of a high taxol containing hybrid *Taxus* species (*Taxus* × *media*) revealed involvement of succinylation in multiple metabolic pathways

Chenjia Shen[1], Jie Xue[1], Tao Sun[1], Hong Guo[1], Lei Zhang[2], Yijun Meng[1,*], Huizhong Wang[1,*]

[1]College of Life and Environmental Sciences, Hangzhou Normal University, Hangzhou 310036, China.

[2]Department of Plant Pathology, North Carolina State University, Raleigh, North Carolina 27607

Yijun Meng

Email: mengyijun@zju.edu.cn

Huizhong Wang:

Email: whz62@163.com

Fig. S1

a

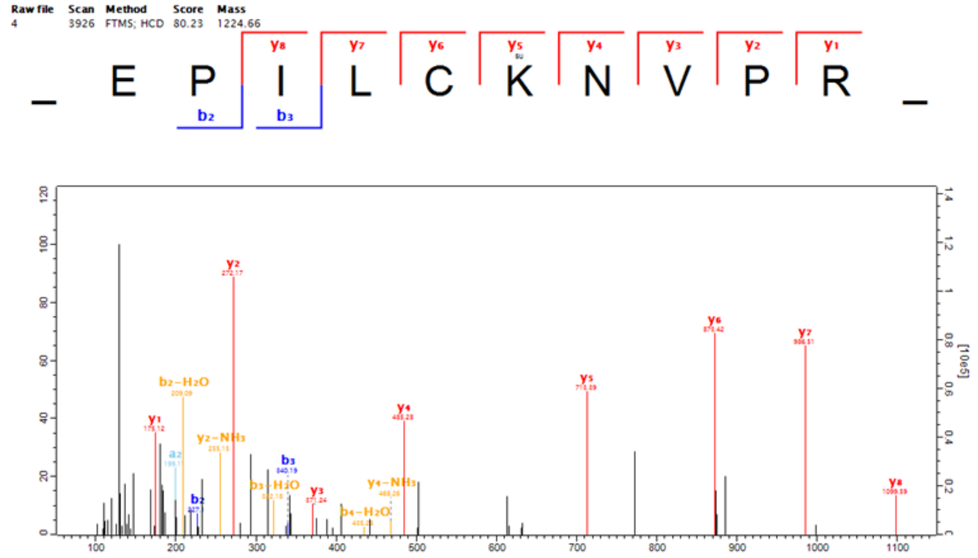

b

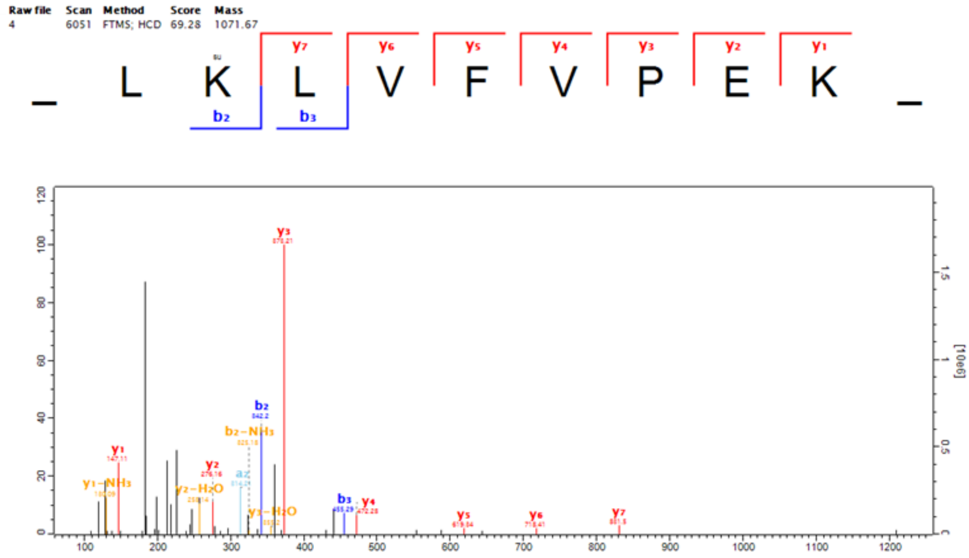

Fig.S1: **Representative MS/MS spectra of succinyl peptides from TCA cycle related enzyme IDH1 (Contig24602).** (a) Succinyl-peptide _EPILCK(su)NVPR_ with succinylation site at K117 in TCA cycle related enzyme IDH1. (b) Succinyl-peptide _LK(su)LVFVPEK_ with succinylation site at K155 in TCA cycle related enzyme IDH1.

Fig. S2

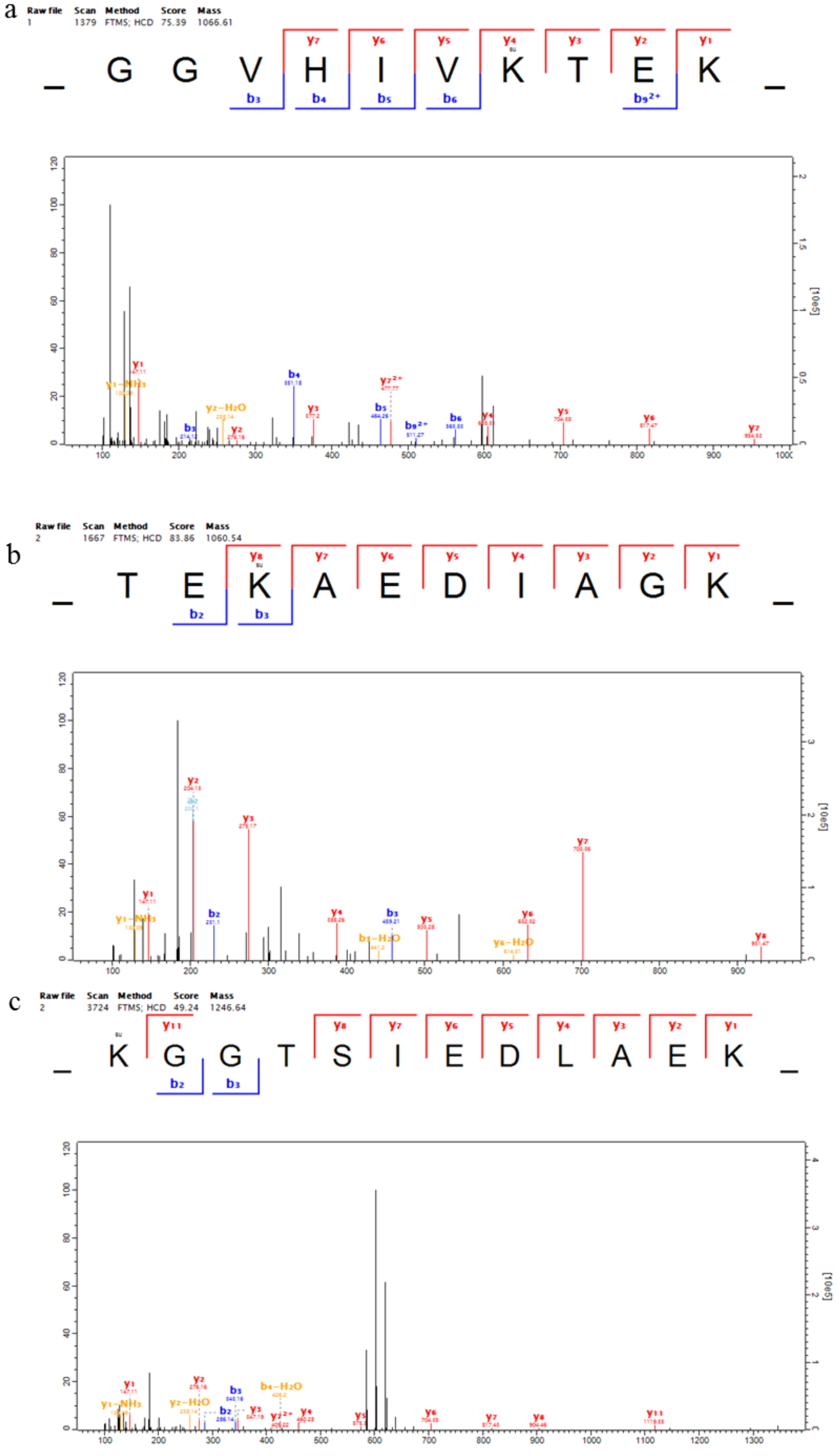
a
Raw file Scan Method Score Mass
1 1379 FTMS; HCD 75.39 1066.61
_ G G V H I V K T E K _
b
Raw file Scan Method Score Mass
2 1667 FTMS; HCD 83.86 1060.54
_ T E K A E D I A G K _
c
Raw file Scan Method Score Mass
2 3724 FTMS; HCD 49.24 1246.64
_ K G G T S I E D L A E K _
[10e5]

d

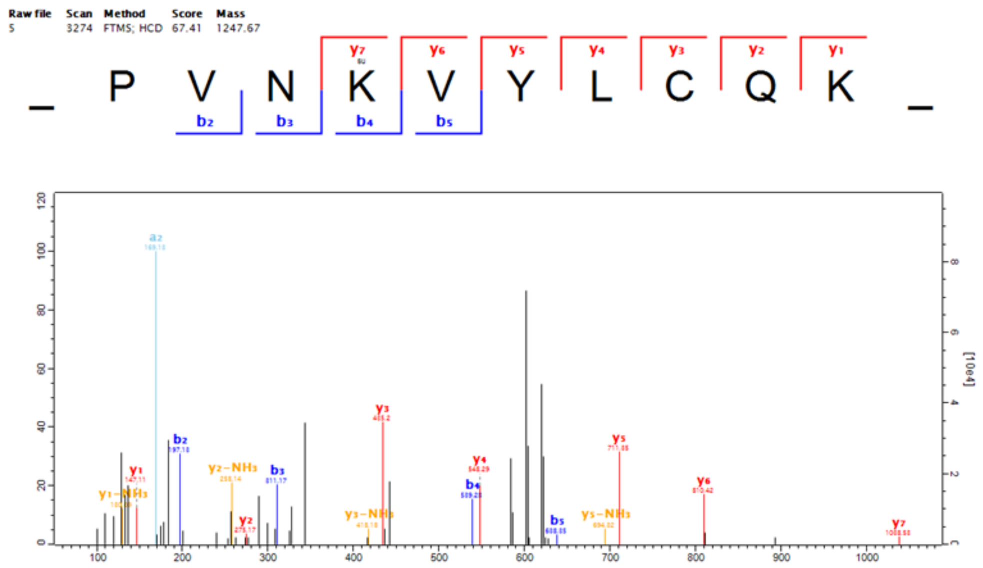

e

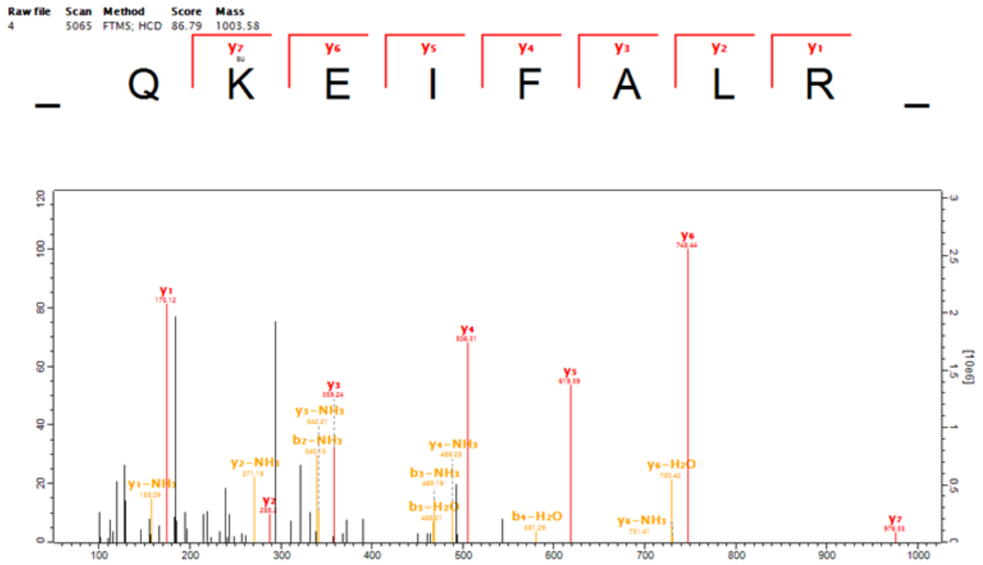

Fig.S2: Representative MS/MS spectra of succinyl peptides from TCA cycle related enzyme LSC1 (Contig4446). (a) Succinyl-peptide _GGVHIVK(su)TEK_ with succinylation site at K97 in TCA cycle related enzyme LSC1. (b) Succinyl-peptide _TEK(su)AEDIAGK_ with succinylation site at K100 in TCA cycle related enzyme LSC1. (c) Succinyl-peptide _K(su)GGTSIEDLAEK_ with succinylation site at K160 in TCA cycle related enzyme LSC1. (d) Succinyl-peptide _PVNK(su)VYLCQK_ with succinylation site at K127 in TCA cycle related enzyme LSC1. (e) Succinyl-peptide _QK(su)EIFALR_ with succinylation site at K259 in TCA cycle related enzyme LSC1.

Fig. S3

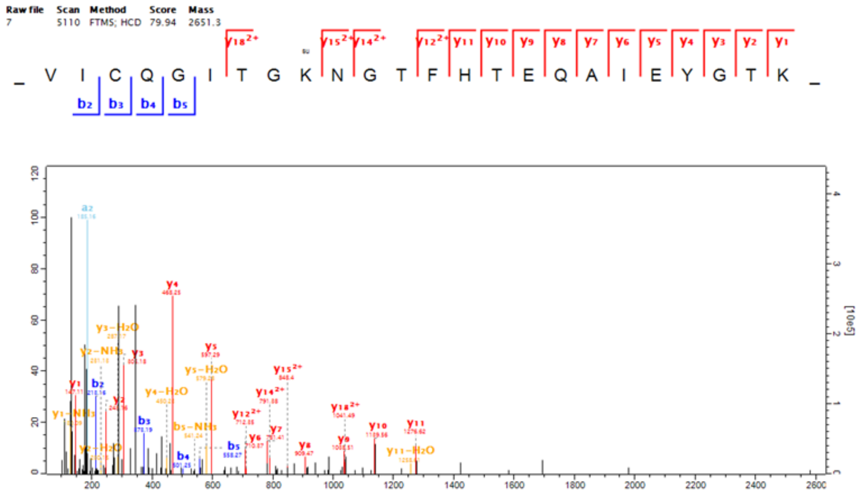

Fig.S3: Representative MS/MS spectra of succinyl peptides from TCA cycle related enzyme LSC2 (Contig18557). Succinyl-peptide _VICQGITGK(su)NGTFHTEQAIEYGTK_ with succinylation site at K55 in TCA cycle related enzyme LSC2.

Fig. S4

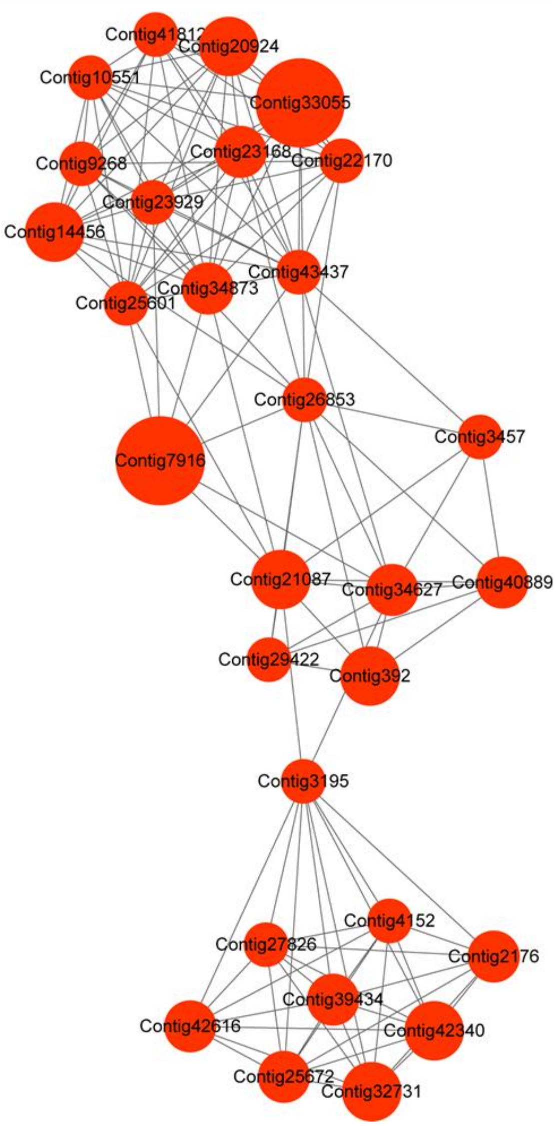

Fig.S4: **Top seven clusters of highly interconnected lysine-succinylated protein networks**. Interaction network of lysine-succinylated oxidative phosphorylation proteins (listed in gene names) was analyzed by the Cytoscape software (version 3.0.1).

Fig. S5

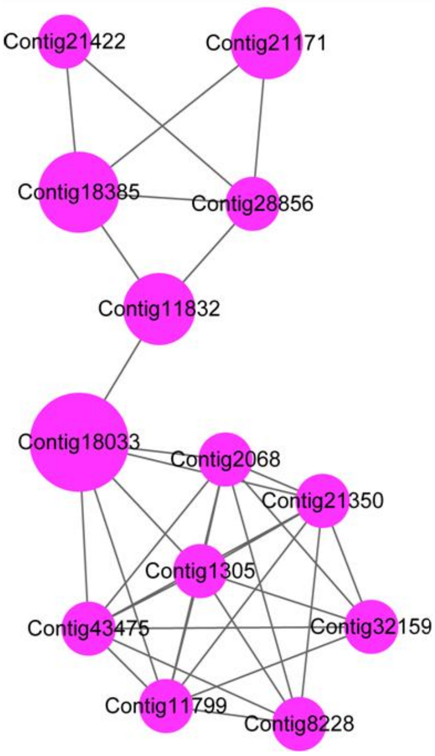

Fig.S5: Top seven clusters of highly interconnected lysine-succinylated protein networks. Interaction network of lysine-succinylated photosynthesis-associated proteins (listed in gene names) was analyzed by the Cytoscape software (version 3.0.1).

Fig. S6

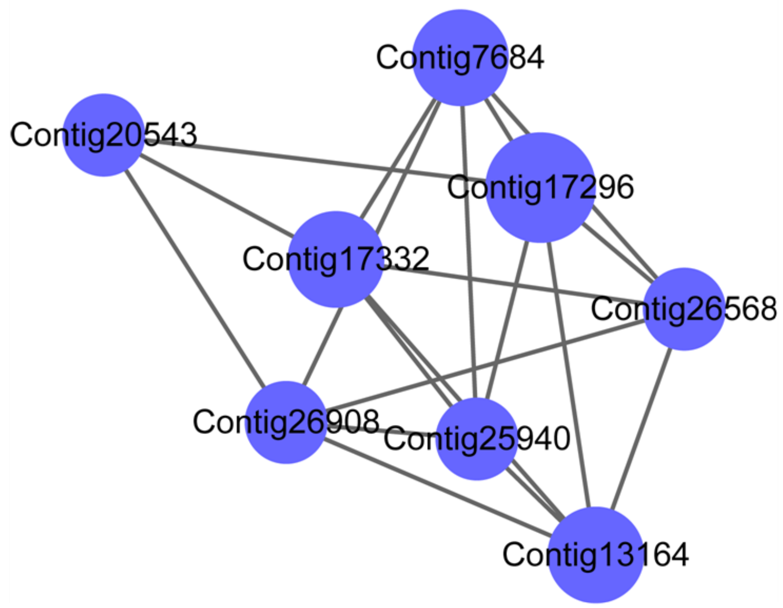

Fig.S6: Top seven clusters of highly interconnected lysine-succinylated protein networks. Interaction network of lysine-succinylated RNA degradation-related proteins (listed in gene names) was analyzed by the Cytoscape software (version 3.0.1).

Fig. S7

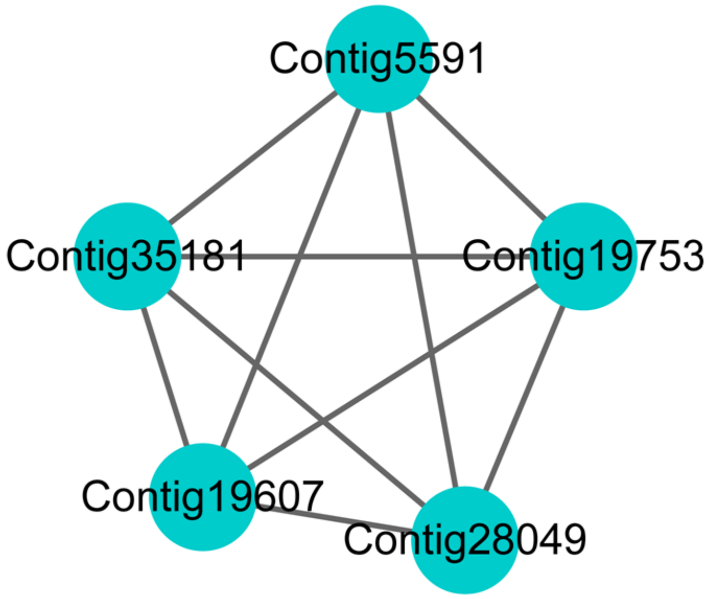

Fig.S7: **Top seven clusters of highly interconnected lysine-succinylated protein networks.** Interaction network of lysine-succinylated ribosome-associated proteins (listed in gene names) was analyzed by the Cytoscape software (version 3.0.1).

Fig. S8

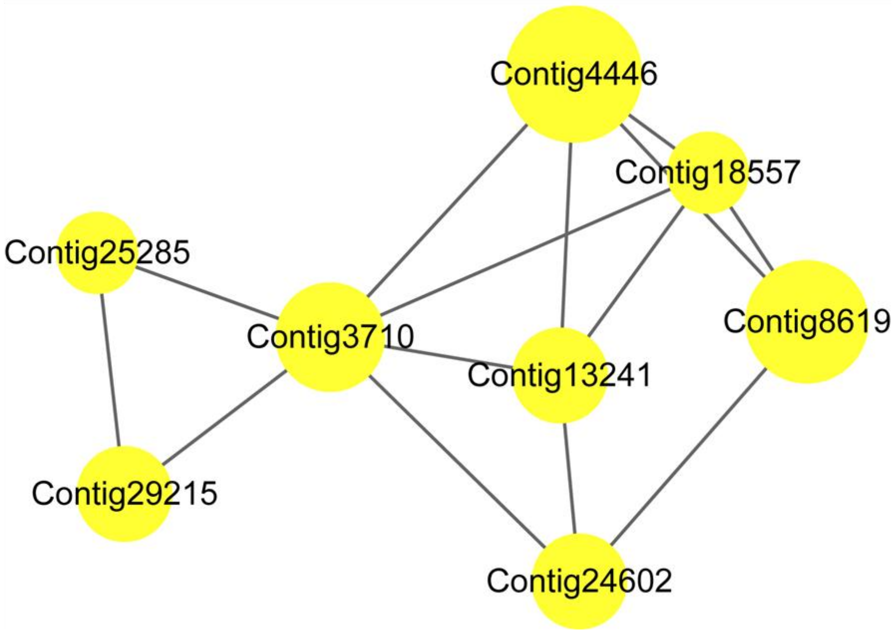

Fig.S8: Top seven clusters of highly interconnected lysine-succinylated protein networks. Interaction network of lysine-succinylated TCA cycle-related proteins (listed in gene names) was analyzed by the Cytoscape software (version 3.0.1).

Table S1. The information of succinylated peptides from histone proteins.

Protein	Localization prob	PEP	Modified sequence	Modification (K) Probabilities	Position	Mass error [ppm]	Peptide Score
Contig22504	1	0.00180931	_EIAQDFK(su)TDLR_	EIAQDFK(1)TDLR	79	-2.958	66.267

Table S2. The information of succinylated sites in each succinylated protein.

Protein	Localization prob	PEP	Modified sequence	Modification (K) Probabilities	Position	Mass error [ppm]	Peptide Score
Contig15874	1	0.0270453	_RPQAK(su)AHFAK_	RPQAK(1)AHFAK	499	0.23561	63.426
Contig20543	1	0.000000138	_AVEQK(su)QVAQQEAER_	AVEQK(1)QVAQQEAER	190	0.48491	103.76
Contig5591	1	0.00184586	_LIFAGK(su)QLEDGR_	LIFAGK(1)QLEDGR	48	0.02452	63.091
Contig10551	1	0.0000989	_TVTLEQK(su)IDR_	TVTLEQK(1)IDR	206	0.47231	113.93
Contig10651	1	0.00544328	_K(su)FLLNIR_	K(1)FLLNIR	731	-1.184	88.643
Contig10828	1	0.000457311	_GDVNSITVGSGTNIQDNTLVHVAK(su)SNLGGK_	GDVNSITVGSGTNIQDNTLVHVAK(1)SNLGGK	110	1.0198	77.149
Contig11799	1	0.0227411	_ESK(su)AFQK_	ESK(1)AFQK	87	-0.061967	64.803
Contig11832	1	0.000525726	_IAGVYNPK(su)R_	IAGVYNPK(1)R	181	-0.34687	103.74
Contig11832	1	0.0000558	_ISFTK(su)EETEYLTNR_	ISFTK(1)EETEYLTNR	237	1.8414	76.759
Contig11832	0.893329	0.0000683	_LKK(su)ELQGSIDK_	LK(0.107)K(0.893)ELQGSIDK	338	-0.087269	111.63
Contig11938	0.956398	0.0254542	_EEPSEIK(su)DLKR_	EEPSEIK(0.956)DLK(0.044)R	98	0.25321	60.255
Contig12149	1	0.00202831	_FK(su)DEIIPVETK_	FK(1)DEIIPVETK	233	-0.94408	65.179
Contig12149	1	0.00798226	_LVDPK(su)TGDEK_	LVDPK(1)TGDEK	247	-0.5309	64.82
Contig6895	1	0.0323334	_K(su)MGWDEK_	K(1)MGWDEK	147	-0.41556	58.815
Contig12178	1	0.00633007	_YQVK(su)GIDNLR_	YQVK(1)GIDNLR	494	0.30324	63.624
Contig12483	1	0.00281111	_LK(su)TPVENSLR_	LK(1)TPVENSLR	382	0.69594	69.346
Contig8119	1	0.0172718	_DPSFSK(su)IEPR_	DPSFSK(1)IEPR	110	-0.9893	55.721
Contig12663	1	0.0175124	_TLK(su)EVQIIPPNVPYR_	TLK(1)EVQIIPPNVPYR	30	1.0317	47.037
Contig5689	1	0.00000677	_IMALDLPHGGHLSHGYQTDTK(su)K_	IMALDLPHGGHLSHGYQTDTK(1)K	191	1.0978	69.03
Contig5689	1	0.0349279	_VLELAHIAANK(su)NTVPGDVSAMVPGGIR_	VLELAHIAANK(1)NTVPGDVSAMVPGGIR	418	0.79412	49.765
Contig1305	1	0.00198227	_LYVCK(su)AQAGDK_	LYVCK(1)AQAGDK	204	-0.10256	67.08
Contig13164	1	0.0288178	_LNSGK(su)VVAVGPGAR_	LNSGK(1)VVAVGPGAR	40	1.6575	51.726
Contig13164	1	0.000932214	_NK(su)EGNIIPVSLK_	NK(1)EGNIIPVSLK	51	-1.8788	70.438
Contig13241	1	0.00859281	_SFEDVK(su)IADR_	SFEDVK(1)IADR	540	0.68534	62.466
Contig13241	1	0.00042893	_YAPTAK(su)DLASR_	YAPTAK(1)DLASR	324	-0.84453	80.014
Contig13277	1	0.0006158	_AVAVLK(su)GNSK_	AVAVLK(1)GNSK	17	-0.28201	92.538
Contig13371	1	0.0272723	_FYIK(su)FYDVK_	FYIK(1)FYDVK	107	-0.49673	49.06
Contig13439	1	0.00113532	_AAALNPVDYK(su)IR_	AAALNPVDYK(1)IR	46	-1.6047	68.536
Contig13439	1	0.000127526	_EAFK(su)HLESGR_	EAFK(1)HLESGR	294	-0.12298	107.58
Contig136	1	0.00187171	_LLK(su)QEPR_	LLK(1)QEPR	19	0.25747	117.93
Contig136	1	0.0000534	_NIANMVPPYQK(su)K_	NIANMVPPYQK(1)K	132	0.025835	75.416
Contig18385	1	0.0149711	_AYEK(su)GISK_	AYEK(1)GISK	92	-0.52004	66.351
Contig18385	1	0.00669624	_EYVWK(su)TLK_	EYVWK(1)TLK	238	-0.30231	73.665
Contig18385	1	0.000550155	_TLK(su)SGQVVPGYGHGVLR_	TLK(1)SGQVVPGYGHGVLR	241	0.98432	65.038
Contig18385	1	7.23E-08	_VK(su)NPWPNVDAHSGVLLNHFGLTEDR_	VK(1)NPWPNVDAHSGVLLNHFGLTEDR	299	-1.5567	84.602
Contig14144	1	0.0267531	_FLM(ox)K(su)TLQQLGSK_	FLMK(1)TLQQLGSK	299	0.53642	49.978

Contig14144	1	0.000984057	_LSEEEASK(su)LR_	LSEEEASK(1)LR	88	1.3303	82.417
Contig1425	1	0.000128754	_ALCTGEK(su)GFGYK_	ALCTGEK(1)GFGYK	141	0.31425	77.062
Contig1425	1	0.019716	_GFGYK(su)GSSFHR_	GFGYK(1)GSSFHR	146	-0.50662	53.567
Contig1425	0.873243	0.0325407	_K(su)TCKIVDSGELP_	K(0.873)TCK(0.127)IVDSGELP	252	0.13763	55.261
Contig14376	0.998537	0.0173981	_VVAVSK(su)TKPVSLIR_	VVAVSK(0.999)TK(0.001)PVSLIR	41	0.63002	65.895
Contig14456	1	0.000112126	_GDLK(su)TDHYLPIHR_	GDLK(1)TDHYLPIHR	196	1.3613	71.558
Contig14456	1	0.000137282	_GVQK(su)VLQNYK_	GVQK(1)VLQNYK	465	0.20368	105.1
Contig14456	1	0.028331	_IMNVIGEPIDEK(su)GDLK_	IMNVIGEPIDEK(1)GDLK	192	1.6488	44.045
Contig17296	1	0.0174825	_SIEFK(su)DR_	SIEFK(1)DR	95	0.37882	67.903
Contig17296	1	0.00998246	_VCAIK(su)APGFGENR_	VCAIK(1)APGFGENR	309	0.29695	57.106
Contig17296	1	0.0237986	_VTMGPK(su)GR_	VTMGPK(1)GR	66	-3.6816	60.478
Contig14654	1	0.0131241	_DVDFDK(su)IAR_	DVDFDK(1)IAR	67	-1.0259	62.463
Contig15015	1	0.017495	_LVFVTNNSTK(su)SR_	LVFVTNNSTK(1)SR	59	-0.74997	53.448
Contig3575	1	0.0342779	_VEGGWK(su)LDGQK_	VEGGWK(1)LDGQK	186	1.3853	45.911
Contig15471	1	0.010187	_AYPK(su)AFVR_	AYPK(1)AFVR	144	0.82018	69.998
Contig15471	1	0.00140028	_EGHK(su)SPGYYDGR_	EGHK(1)SPGYYDGR	105	-0.46279	66.326
Contig15471	1	0.00459279	_QIDYLLK(su)NK_	QIDYLLK(1)NK	84	-0.34034	73.573
Contig15674	1	0.00483808	_DDFIEK(su)DR_	DDFIEK(1)DR	356	-1.6078	80.165
Contig15674	0.999951	0.00406678	_DKLNK(su)YGR_	DKLNK(1)YGR	164	-0.61858	95.531
Contig15674	1	0.010357	_GHYLNATAGTCEEMMK(su)R_	GHYLNATAGTCEEMMK(1)R	252	-0.099932	41.088
Contig15674	1	0.00549276	_LGLSAK(su)NYGR_	LGLSAK(1)NYGR	183	-1.1725	67.334
Contig15674	1	0.030797	_PK(su)LGLSAK_	PK(1)LGLSAK	177	-0.82252	57.859
Contig15674	1	0.00744994	_TETK(su)ASVGFK_	TETK(1)ASVGFK	8	0.09427	66.056
Contig15684	1	0.00764879	_EIEK(su)DLGR_	EIEK(1)DLGR	141	-0.29744	73.26
Contig15684	1	0.0175021	_SFLGK(su)ICGR_	SFLGK(1)ICGR	477	1.2169	59.35
Contig15732	1	0.0334188	_AIK(su)YYCELR_	AIK(1)YYCELR	203	-0.40158	46.001
Contig15732	1	0.00118064	_SENLEK(su)QCLAK_	SENLEK(1)QCLAK	195	0.77881	70.942
Contig7396	1	0.00890464	_LEAK(su)TVYGALR_	LEAK(1)TVYGALR	160	2.7851	61.344
Contig2798	1	0.000690841	_AQVLDSEGK(su)R_	AQVLDSEGK(1)R	185	0.32761	85.355
Contig2798	1	0.0291164	_IVPEK(su)IAYVVER_	IVPEK(1)IAYVVER	19	-1.0764	45.28
Contig15920	1	0.0296311	_FVFYDEK(su)K_	FVFYDEK(1)K	236	1.0816	44.349
Contig1604	1	0.00193186	_K(su)LEPTPGDMVR_	K(1)LEPTPGDMVR	335	3.1715	63.624
Contig25574	1	0.0182355	_EDLLAK(su)PENIR_	EDLLAK(1)PENIR	698	0.72888	53.237
Contig16402	1	0.00197345	_ETLQQNK(su)ILK_	ETLQQNK(1)ILK	382	-0.44736	73.233
Contig16402	1	0.00287083	_VEK(su)VVVSDR_	VEK(1)VVVSDR	560	0.12013	83.883
Contig19420	1	0.00181613	_EVAQQIESINK(su)K_	EVAQQIESINK(1)K	44	0.615	58.172
Contig19420	1	0.0106746	_YHVGK(su)IGK_	YHVGK(1)IGK	89	0.95394	70.69

Contig16825	1	0.00000718	_DGPADTTALAK(su)WR_	DGPADTTALAK(1)WR	120	-0.42387	96.745
Contig16825	1	0.00255909	_WAFK(su)LLQR_	WAFK(1)LLQR	302	-0.60582	91.626
Contig39307	1	0.00090625	_MEK(su)FYWAPTR_	MEK(1)FYWAPTR	309	1.3513	79.659
Contig19420	1	1.82E-08	_AASANPNK(su)LHIGDVAIPR_	AASANPNK(1)LHIGDVAIPR	136	-0.1539	97.235
Contig19420	1	0.0253824	_CLEQQPK(su)IK_	CLEQQPK(1)IK	248	-0.68899	51.949
Contig16895	1	0.000233179	_EVAQQIK(su)SINK_	EVAQQIK(1)SINK	41	0.76878	85.813
Contig19420	1	0.0000363	_HALSAVGEMFK(su)R_	HALSAVGEMFK(1)R	338	-0.2622	81.92
Contig19420	1	0.00017768	_IEK(su)GSSANIYVNNEAYR_	IEK(1)GSSANIYVNNEAYR	254	0.63967	68.576
Contig19420	1	0.0183306	_IENVELDK(su)CLGK_	IENVELDK(1)CLGK	234	-0.4337	51.726
Contig19420	1	0.0000473	_SEK(su)NM(ox)AFLALR_	SEK(1)NMAFLALR	295	-1.091	105.98
Contig16920	1	0.0191136	_NDNNIVLK(su)TEGK_	NDNNIVLK(1)TEGK	281	0.94722	53.034
Contig30441	1	0.000272558	_GQLEEEK(su)GGIK_	GQLEEEK(1)GGIK	282	-0.46078	86.014
Contig17332	1	0.0020169	_ALTYPVK(su)LIAK_	ALTYPVK(1)LIAK	523	0.29923	67.704
Contig17332	1	0.00284302	_LAAK(su)VDAIR_	LAAK(1)VDAIR	494	0.65631	85.862
Contig454	1	0.0252385	_GK(su)FDESR_	GK(1)FDESR	12	-1.3177	62.659
Contig43032	1	0.00992143	_SVITALK(su)R_	SVITALK(1)R	264	-0.45627	73.248
Contig35544	1	0.0128083	_TSVDILK(su)VGGYK_	TSVDILK(1)VGGYK	160	0.55484	58.172
Contig17831	1	0.0236984	_AHIAGLHDK(su)GK_	AHIAGLHDK(1)GK	26	-0.034842	54.198
Contig17880	1	0.0106531	_ASHLLIK(su)HQGSR_	ASHLLIK(1)HQGSR	14	0.55496	58.098
Contig17946	1	0.0362697	_YYM(ox)LGGK(su)GGVGK_	YYMLGGK(1)GGVGK	11	-1.0798	46.796
Contig18033	1	0.000129531	_GADLQVLSAK(su)LAADK_	GADLQVLSAK(1)LAADK	129	2.3846	74.296
Contig18033	1	0.0213239	_ILLEDVK(su)K_	ILLEDVK(1)K	52	0.59737	58.981
Contig18033	1	0.00101746	_LAVEAWGLK(su)NCTQR_	LAVEAWGLK(1)NCTQR	295	-0.66078	64.104
Contig18033	0.999416	0.0191489	_SPAVPALTK(su)ILLEDVKK_	SPAVPALTK(0.999)ILLEDVK(0.001)K	45	0.28897	63.374
Contig18033	1	0.00180809	_VAGK(su)VFR_	VAGK(1)VFR	344	0.40594	121.68
Contig18033	1	0.0376553	_VK(su)AVCIVHNETATGVTNNLAAVR_	VK(1)AVCIVHNETATGVTNNLAAVR	139	-1.4083	49.66
Contig18104	1	0.00858278	_QLSYTFK(su)R_	QLSYTFK(1)R	290	-0.66135	71.501
Contig24498	1	0.0000746	_YLLYK(su)DVNR_	YLLYK(1)DVNR	280	0.52585	117.89
Contig18557	1	0.0000193	_VICQGITGK(su)NGTFHTEQAIEYGTK_	VICQGITGK(1)NGTFHTEQAIEYGTK	55	-0.89348	79.942
Contig18880	1	0.00360583	_SPNFAGVK(su)ECVGNDR_	SPNFAGVK(1)ECVGNDR	216	-0.62904	57.288
Contig1890	1	0.000856413	_DYNPEQNK(su)DNVLAR_	DYNPEQNK(1)DNVLAR	22	1.3228	64.104
Contig19607	1	0.000689917	_VK(su)FLDDQNR_	VK(1)FLDDQNR	29	1.062	101.65
Contig19663	1	0.000575297	_TTFLSDPEVK(su)R_	TTFLSDPEVK(1)R	173	0.84657	72.531
Contig19753	1	0.00823713	_IIK(su)VTGPR_	IIK(1)VTGPR	26	-0.016043	73.665
Contig2068	1	0.00188303	_SK(su)AGIPSLPR_	SK(1)AGIPSLPR	280	-0.34661	78.342
Contig2087	1	0.0157808	_NTGVK(su)PEILK_	NTGVK(1)PEILK	591	1.4251	58.743
Contig2087	1	2.89E-15	_NVGK(su)AITSLPETFNPHR_	NVGK(1)AITSLPETFNPHR	600	-0.39182	121.99

Contig20924	1	0.00452089	_FDALLK(su)EAK_	FDALLK(1)EAK	105	1.2834	76.282
Contig20924	1	0.00339869	_K(su)EFLNLR_	K(1)EFLNLR	32	0.85883	111.04
Contig20924	1	0.00520107	_TYEEVK(su)R_	TYEEVK(1)R	45	0.61639	91.906
Contig21087	1	0.000334213	_GVVESLK(su)IITR_	GVVESLK(1)IITR	178	3.2261	83.045
Contig21087	1	0.0131891	_K(su)TDGLFLQCCR_	K(1)TDGLFLQCCR	215	-1.0916	47.894
Contig21087	1	0.00150885	_VAEYAFHYAK(su)THGR_	VAEYAFHYAK(1)THGR	197	1.269	62.162
Contig21171	1	1.75E-08	_GLSLIGEDVK(su)ILAR_	GLSLIGEDVK(1)ILAR	476	0.27444	111.79
Contig21171	1	9.25E-08	_LNAIQEASGGK(su)R_	LNAIQEASGGK(1)R	406	0.49644	125.97
Contig21171	1	0.00518624	_VDK(su)LMELR_	VDK(1)LMELR	388	-0.23666	79.893
Contig21350	1	0.0331093	_ASK(su)QVYNISGSR_	ASK(1)QVYNISGSR	287	1.2556	45.28
Contig21422	1	0.00000066	_SLSAISAEVK(su)ELADK_	SLSAISAEVK(1)ELADK	411	-0.34938	96.673
Contig21609	1	0.000961914	_AVVAK(su)NLQNR_	AVVAK(1)NLQNR	240	0.59434	83.948
Contig21709	1	0.0356247	_SIPTVMLFK(su)SGEK_	SIPTVMLFK(1)SGEK	163	0.27797	42.317
Contig2176	1	0.00112255	_VIK(su)VVSDR_	VIK(1)VVSDR	88	-0.51995	113.26
Contig2176	1	0.0209655	_VLDQK(su)FGIIK_	VLDQK(1)FGIIK	181	0.29048	53.751
Contig21854	1	0.00659187	_IAGLEVK(su)R_	IAGLEVK(1)R	218	-0.79547	81.296
Contig21943	1	0.0186404	_YLAEFK(su)TGNER_	YLAEFK(1)TGNER	139	1.4202	52.247
Contig22424	1	0.00310806	_ASGLK(su)PIVDDDK_	ASGLK(1)PIVDDDK	537	-1.0379	78.848
Contig22170	1	0.0033155	_LAK(su)VDLGR_	LAK(1)VDLGR	526	-0.79023	91.318
Contig2229	1	0.0179288	_FTEK(su)AIK_	FTEK(1)AIK	99	0.076195	68.915
Contig22408	1	0.00859537	_DHIVK(su)GILIGLR_	DHIVK(1)GILIGLR	470	-0.92488	59.265
Contig22504	1	0.00180931	_EIAQDFK(su)TDLR_	EIAQDFK(1)TDLR	80	-2.958	66.267
Contig22710	1	0.00409923	_GK(su)GVYQFTNK_	GK(1)GVYQFTNK	72	0.3524	66.92
Contig23147	1	0.000301429	_PGTFK(su)QTTLCIR_	PGTFK(1)QTTLCIR	285	0.33427	72.29
Contig23168	1	0.0102512	_AMK(su)QVAGK_	AMK(1)QVAGK	159	-1.1602	72.789
Contig23168	1	0.0344815	_VGSAAQMK(su)AM(ox)K_	VGSAAQMK(1)AMK	156	-1.0789	50.353
Contig23293	1	0.00000334	_VCAAALNPVDFK(su)R_	VCAAALNPVDFK(1)R	91	-0.71718	92.856
Contig6210	1	0.01398	_MVLLK(su)GVDER_	MVLLK(1)GVDER	384	0.48049	58.699
Contig23896	1	0.0222101	_LSK(su)VLDVYEER_	LSK(1)VLDVYEER	143	1.1305	49.448
Contig23929	1	0.00186468	_HEEIVDLAK(su)K_	HEEIVDLAK(1)K	295	-0.52568	72.607
Contig23978	1	0.017878	_VVVIK(su)ELK_	VVVIK(1)ELK	96	-0.93199	62.546
Contig26568	1	0.0000288	_TLAK(su)VVGSEEEAK_	TLAK(1)VVGSEEEAK	23	0.47588	89.231
Contig24406	1	0.0333016	_MEGK(su)LTGSGHSIAAAR_	MEGK(1)LTGSGHSIAAAR	778	-0.84155	46.892
Contig24557	1	0.000121008	_HYIQDPPTVEK(su)CHELSK_	HYIQDPPTVEK(1)CHELSK	54	0.038971	66.826
Contig24602	1	0.00102436	_EPILCK(su)NVPR_	EPILCK(1)NVPR	117	1.4245	80.231
Contig24602	1	0.00599536	_LK(su)LVFVPEK_	LK(1)LVFVPEK	155	0.043552	69.275
Contig24949	1	0.00115507	_LEQETLK(su)WCR_	LEQETLK(1)WCR	270	-1.3475	74.841

Contig2510	1	0.00239228	_TASK(su)SVSSSPWYGPDR_	TASK(1)SVSSSPWYGPDR	46	0.72123	63.682
Contig25285	1	0.0000279	_ELAK(su)EGINAEVINLR_	ELAK(1)EGINAEVINLR	272	-0.03999	85.909
Contig3062	1	0.000468099	_IISSIEQK(su)EESR_	IISSIEQK(1)EESR	77	-0.11957	71.176
Contig25407	1	0.000760316	_EK(su)GIFTNVTSPSAK_	EK(1)GIFTNVTSPSAK	287	-0.9437	69.261
Contig25407	1	0.00998358	_TIAFK(su)VR_	TIAFK(1)VR	90	0.65188	76.847
Contig25407	1	0.00158226	_TQVAYGSK(su)NEIIR_	TQVAYGSK(1)NEIIR	348	0.091215	61.409
Contig25407	1	0.00324284	_VIK(su)NVDDR_	VIK(1)NVDDR	335	0.3037	89.698
Contig25601	1	0.00147889	_GLCGGFNNMVLK(su)R_	GLCGGFNNMVLK(1)R	143	-0.46222	57.175
Contig25672	1	6.94E-11	_GILAM(ox)DESNATCGK(su)R_	GILAMDESNATCGK(1)R	412	-0.3808	111.12
Contig25672	1	0.000669105	_TAK(su)TVASPGR_	TAK(1)TVASPGR	391	-0.40179	91.658
Contig25847	1	0.0167421	_ILDVEQCK(su)ELK_	ILDVEQCK(1)ELK	258	0.69626	52.391
Contig25847	1	0.0337507	_K(su)NEVFTK_	K(1)NEVFTK	113	0.55173	58.917
Contig25868	1	0.000439528	_EVANK(su)LER_	EVANK(1)LER	188	-1.9549	123.98
Contig25868	1	0.0000962	_IENVGLDK(su)CLGR_	IENVGLDK(1)CLGR	234	-1.2386	79.492
Contig25940	1	0.00172361	_NDAVGTIK(su)HVEK_	NDAVGTIK(1)HVEK	232	-0.16874	64.827
Contig26084	1	0.0242014	_TPPLK(su)LSK_	TPPLK(1)LSK	93	-3.2702	59.368
Contig26567	1	5.07E-08	_SMTQHNVGALLVVK(su)PGAEK_	SMTQHNVGALLVVK(1)PGAEK	98	1.1012	91.589
Contig26788	1	0.0282201	_DGVYPEK(su)ANPGR_	DGVYPEK(1)ANPGR	165	-0.86203	48.568
Contig26788	1	0.0234643	_FK(su)INYQFYR_	FK(1)INYQFYR	138	-0.076226	49.715
Contig26853	1	0.0173166	_VPAEVIESIK(su)R_	VPAEVIESIK(1)R	101	-1.52	50.354
Contig26908	1	0.00185238	_VLVTDQK(su)ISTIK_	VLVTDQK(1)ISTIK	242	-0.54436	63.091
Contig27115	1	0.00467872	_LK(su)HGEALVQNEGDSDISK_	LK(1)HGEALVQNEGDSDISK	38	0.5175	60.062
Contig27115	1	0.0235194	_LPK(su)NVVYR_	LPK(1)NVVYR	146	-1.5083	57.177
Contig27136	1	0.0000199	_SLFK(su)GAGANILR_	SLFK(1)GAGANILR	288	0.039803	94.465
Contig27136	1	0.00000972	_TIK(su)SDGVAGLYR_	TIK(1)SDGVAGLYR	184	-0.62128	103.21
Contig27136	1	0.0169239	_VK(su)LLIQNQDEMLK_	VK(1)LLIQNQDEMLK	49	-0.95739	50.088
Contig36350	1	0.0128339	_SQSPWK(su)R_	SQSPWK(1)R	47	0.090914	73.616
Contig27558	1	0.00791819	_VLSVVK(su)SFPK_	VLSVVK(1)SFPK	58	-0.18507	64.82
Contig27743	1	0.0336938	_VPNTK(su)NLGLEEVGVK_	VPNTK(1)NLGLEEVGVK	361	0.31643	44.252
Contig27826	1	0.00753376	_ALNDHHVLLEGTLLK(su)PNMVTPGSDAPK_	ALNDHHVLLEGTLLK(1)PNMVTPGSDAPK	225	2.3247	60.95
Contig30157	1	0.000177313	_LVAQK(su)GLHLIR_	LVAQK(1)GLHLIR	449	-1.536	88.495
Contig28049	1	0.0105286	_AMK(su)FSVSPVVR_	AMK(1)FSVSPVVR	482	1.0906	60.255
Contig28157	1	0.0244983	_GYVASDSK(su)NDPAK_	GYVASDSK(1)NDPAK	318	0.21781	51.726
Contig28157	1	0.000537363	_QTVAVGVIK(su)AVEK_	QTVAVGVIK(1)AVEK	427	0.16708	72.321
Contig28177	1	0.0312374	_EPAGEDRPSDK(su)PR_	EPAGEDRPSDK(1)PR	59	0.74976	55.261
Contig28454	1	0.028645	_ELK(su)LDLEK_	ELK(1)LDLEK	458	0.25004	54.722
Contig28856	1	0.0000388	_TALSDEYFVK(su)R_	TALSDEYFVK(1)R	409	-0.49339	108.7

Contig29215	1	0.0364627	_EFEK(su)EAK_	EFEK(1)EAK	343	0.20248	56.122
Contig29215	1	0.003042	_SPAYYK(su)R_	SPAYYK(1)R	248	-0.64209	114.02
Contig32342	1	0.01046	_ESGLYGICGDK(su)DMR_	ESGLYGICGDK(1)DMR	260	-1.7407	53.3
Contig29422	1	0.009417	_IFLEDGHQIACAQSFAK(su)NM(ox)GLYGQR_	IFLEDGHQIACAQSFAK(1)NMGLYGQR	271	-1.1268	60.735
Contig29951	1	0.00533772	_DDELLATDTK(su)SVPR_	DDELLATDTK(1)SVPR	202	0.73762	56.569
Contig29956	1	0.00670255	_IAGLDVK(su)R_	IAGLDVK(1)R	223	-0.43538	81.296
Contig29956	1	0.00151141	_NPK(su)VIENSEGAR_	NPK(1)VIENSEGAR	82	0.78357	66.267
Contig29956	1	0.0296317	_SCLK(su)DAGVTVK_	SCLK(1)DAGVTVK	376	-1.7072	49.448
Contig29956	1	0.000104096	_SESIDLSK(su)DR_	SESIDLSK(1)DR	305	-0.43938	113.93
Contig29956	1	0.000995617	_TKEPCK(su)SCLK_	TKEPCK(1)SCLK	372	-0.85225	91.549
Contig3071	1	0.00768938	_EK(su)VVIPK_	EK(1)VVIPK	419	1.0649	82.671
Contig3071	1	0.0054794	_TCEK(su)SPIPR_	TCEK(1)SPIPR	405	0.45731	70.908
Contig30828	1	0.0110055	_NFHK(su)FLEVR_	NFHK(1)FLEVR	216	-1.5298	61.78
Contig31013	1	0.00192462	_AEFLK(su)AVENNR_	AEFLK(1)AVENNR	673	-0.4666	66.56
Contig31013	1	0.00720821	_IADVVK(su)LR_	IADVVK(1)LR	349	-3.0423	75.738
Contig31013	1	0.0322916	_MYK(su)TLLGK_	MYK(1)TLLGK	394	-0.22008	53.775
Contig9786	1	0.00774013	_VEEISK(su)GVGDK_	VEEISK(1)GVGDK	79	-0.1294	61.932
Contig6895	1	0.00754071	_LNVFK(su)PDK_	LNVFK(1)PDK	169	0.39132	73.573
Contig31735	0.857382	0.00345792	_K(su)NKFVEEWGAAR_	K(0.857)NK(0.143)FVEEWGAAR	9	0.4871	73.632
Contig31735	1	0.000416526	_NK(su)FVEEWGAAR_	NK(1)FVEEWGAAR	11	1.9641	77.744
Contig3195	1	0.0000516	_HVIGEDNQFIGK(su)K_	HVIGEDNQFIGK(1)K	167	0.22772	69.815
Contig32159	1	0.0159958	_TIQDEINDLK(su)K_	TIQDEINDLK(1)K	171	-0.5233	48.948
Contig32510	1	0.00240926	_NIWK(su)VINWK_	NIWK(1)VINWK	210	-0.85972	83.168
Contig32510	1	0.00197665	_NLAPVK(su)EGGGEPPK_	NLAPVK(1)EGGGEPPK	111	0.099055	65.895
Contig32510	1	0.0244972	_NVRPDYLK(su)NIWK_	NVRPDYLK(1)NIWK	206	1.1389	57.401
Contig32572	1	0.000358862	_GLLGK(su)NITER_	GLLGK(1)NITER	89	0.22661	94.692
Contig32572	1	0.000150004	_VLDVYDK(su)R_	VLDVYDK(1)R	150	-0.7542	127.02
Contig32572	1	0.00469617	_YIAQK(su)YETR_	YIAQK(1)YETR	77	0.38432	72.243
Contig38810	1	0.0245236	_GSDLSGK(su)DFSGK_	GSDLSGK(1)DFSGK	109	0.2405	53.448
Contig32590	1	0.00334647	_SLDEVIK(su)R_	SLDEVIK(1)R	460	0.12344	94.114
Contig3264	1	0.0252177	_VEK(su)LGTELK_	VEK(1)LGTELK	67	2.0279	55.084
Contig32731	0.998905	0.00385664	_FYKEEEK(su)NDPEFAK_	FYK(0.001)EEEK(0.999)NDPEFAK	188	0.40361	65.895
Contig32731	0.999962	0.0000872	_GVTPK(su)YSLKPLVPR_	GVTPK(1)YSLKPLVPR	133	0.51806	89.43
Contig32731	1	0.000947636	_YSLK(su)PLVPR_	YSLK(1)PLVPR	137	0.86098	99.442
Contig33055	1	0.00106795	_AMK(su)QVCGSLK_	AMK(1)QVCGSLK	390	-0.92966	83.862
Contig33055	1	0.00233562	_GK(su)LTNEIEMK_	GK(1)LTNEIEMK	493	0.23785	72.2
Contig33055	1	0.00223765	_ITDYSTDLK(su)VDEIGR_	ITDYSTDLK(1)VDEIGR	19	0.21916	59.709

Contig33055	1	0.0214019	_LTEVLK(su)QPQYSPLPIEK_	LTEVLK(1)QPQYSPLPIEK	435	-0.45974	45.384
Contig33055	1	0.0124135	_M(ox)ELTTLLEK(su)R_	MELTTLLEK(1)R	9	-1.3696	53.237
Contig33055	1	0.000176024	_TGSIVDVPVGK(su)ALLGR_	TGSIVDVPVGK(1)ALLGR	96	-0.095752	74.853
Contig33055	1	0.0000433	_VGSAAQLK(su)AM(ox)K_	VGSAAQLK(1)AMK	387	0.065175	113.93
Contig33154	1	0.000989454	_AAAEK(su)IFSAK_	AAAEK(1)IFSAK	113	-0.90527	85.355
Contig33154	1	0.000297036	_AK(su)TLSGPVSSPK_	AK(1)TLSGPVSSPK	39	0.53052	78.324
Contig33154	1	0.000027	_HK(su)EHIAAYGEGNER_	HK(1)EHIAAYGEGNER	279	0.50337	86.498
Contig34414	1	0.00432016	_WAFELLK(su)R_	WAFELLK(1)R	280	1.4333	84.568
Contig3457	1	0.00892758	_VEK(su)HIQDAISK_	VEK(1)HIQDAISK	380	1.152	60.509
Contig34617	1	0.0220906	_NLGIK(su)CR_	NLGIK(1)CR	117	-1.3335	64.803
Contig34627	1	0.00740656	_FK(su)DIFEEEYEK_	FK(1)DIFEEEYEK	287	0.34295	56.205
Contig34627	1	0.00010776	_MTK(su)DLAILVHGSR_	MTK(1)DLAILVHGSR	437	-0.0085083	75.911
Contig34715	1	0.00297136	_LPPK(su)AISAAK_	LPPK(1)AISAAK	400	-0.59393	72.2
Contig34759	1	0.036719	_IK(su)GYPLWPDANK_	IK(1)GYPLWPDANK	357	0.0050462	40.767
Contig34759	1	0.0113868	_LEICTEK(su)K_	LEICTEK(1)K	58	-1.1157	67.897
Contig34759	1	0.00317724	_SQGEIPTTWK(su)R_	SQGEIPTTWK(1)R	237	-0.61905	58.172
Contig34873	1	0.0188736	_ISK(su)AFFR_	ISK(1)AFFR	133	-0.38429	67.344
Contig34873	1	0.00329917	_SIK(su)SIQK_	SIK(1)SIQK	60	0.25603	97.602
Contig35075	1	0.0337975	_INPDSAK(su)GYK_	INPDSAK(1)GYK	203	0.62808	48.981
Contig35181	1	0.0185229	_ITTLVK(su)AANVK_	ITTLVK(1)AANVK	32	-0.95034	56.404
Contig35287	1	0.000364851	_ENNFK(su)QPGER_	ENNFK(1)QPGER	429	0.26119	91.549
Contig35316	1	0.00150181	_THGGIDVIK(su)K_	THGGIDVIK(1)K	267	-0.11532	78.098
Contig39434	1	0.00809697	_GILAVDESNATCGK(su)R_	GILAVDESNATCGK(1)R	78	1.1546	47.037
Contig39434	1	0.00264229	_SAEYYK(su)QGAR_	SAEYYK(1)QGAR	175	0.92745	70.028
Contig35702	1	0.00073221	_GIPTFEK(su)TQQEGK_	GIPTFEK(1)TQQEGK	113	1.2718	67.997
Contig35859	1	0.0000408	_EK(su)AGLYSDADR_	EK(1)AGLYSDADR	103	-0.043962	113.38
Contig35859	1	0.00121575	_EK(su)VTIDPDDPNAVSR_	EK(1)VTIDPDDPNAVSR	79	0.77184	64.52
Contig35859	0.974544	0.0265603	_K(su)EKVTIDPDDPNAVSR_	K(0.975)EK(0.025)VTIDPDDPNAVSR	77	-0.29395	57.197
Contig35859	1	0.0000586	_VK(su)SGLEDHLGAEK_	VK(1)SGLEDHLGAEK	140	0.086687	83.358
Contig35859	1	0.00012527	_YEK(su)QLELDVAK_	YEK(1)QLELDVAK	202	0.62865	88.155
Contig35931	1	0.00817408	_LIVIDYK(su)PMR_	LIVIDYK(1)PMR	109	2.1509	61.161
Contig36475	1	0.0397166	_AEVTASLNQMK(su)K_	AEVTASLNQMK(1)K	170	-0.87177	42.843
Contig36475	1	0.0000953	_LQEEADAILK(su)AAR_	LQEEADAILK(1)AAR	156	-0.0019618	77.185
Contig36589	1	0.0206574	_ACSVDAVK(su)YIR_	ACSVDAVK(1)YIR	103	-0.80136	51.998
Contig3710	1	0.00064571	_DVFLEK(su)HGVK_	DVFLEK(1)HGVK	289	1.0815	78.401
Contig3710	1	0.0227747	_HGVK(su)LGFMSGFIK_	HGVK(1)LGFMSGFIK	293	-0.69386	50.897
Contig3710	1	0.0206653	_LNFAEIEK(su)TMSALAK_	LNFAEIEK(1)TMSALAK	359	-0.58859	48.131

Contig37760	0.999814	0.0232073	_QLEK(su)ELKER_	QLEK(1)ELKER	118	0.70333	71.692
Contig38639	1	0.0214151	_VWK(su)GTAFGGFK_	VWK(1)GTAFGGFK	318	0.7817	53.567
Contig38899	1	0.000124963	_AGFK(su)AIALTVDTPR_	AGFK(1)AIALTVDTPR	150	0.95742	73.834
Contig38899	1	0.000338442	_DVK(su)WLQTITK_	DVK(1)WLQTITK	217	0.63902	92.611
Contig38899	1	0.0186428	_SLSWK(su)DVK_	SLSWK(1)DVK	214	-0.012829	60.91
Contig38987	1	0.00825288	_EFLENLK(su)LGEGVK_	EFLENLK(1)LGEGVK	247	-0.8514	56.569
Contig38987	1	0.00593334	_LM(ox)K(su)VISDSVK_	LMK(1)VISDSVK	127	0.42637	70.808
Contig38987	1	0.000615608	_MSK(su)SGRPIEPR_	MSK(1)SGRPIEPR	332	0.0026157	89.9
Contig392	1	0.00303912	_IFK(su)IGGLGVK_	IFK(1)IGGLGVK	186	0.64485	70.908
Contig392	1	0.00913778	_IGGLGVK(su)TYR_	IGGLGVK(1)TYR	193	0.94022	64.82
Contig392	1	0.00149093	_TEEGK(su)PLILNVVR_	TEEGK(1)PLILNVVR	89	-0.98003	62.338
Contig40889	1	0.00202323	_EIK(su)VECTIPR_	EIK(1)VECTIPR	36	0.26842	72.2
Contig40889	1	0.00215885	_VNAELK(su)TYMTR_	VNAELK(1)TYMTR	367	-0.5619	64.121
Contig4152	1	0.0212049	_IAIAK(su)AGYTGK_	IAIAK(1)AGYTGK	238	0.37842	56.205
Contig41772	1	0.00878111	_GMFDK(su)EK_	GMFDK(1)EK	271	-0.61767	79.146
Contig41772	1	0.0290666	_M(ox)K(su)EGVLIVNNAR_	MK(1)EGVLIVNNAR	278	0.48062	49.448
Contig41772	1	0.0258777	_VVGVFYK(su)ANEYASK_	VVGVFYK(1)ANEYASK	42	-0.20886	47.037
Contig41772	1	0.0123656	_YAAGVK(su)DMLDK_	YAAGVK(1)DMLDK	353	-0.86025	59.265
Contig41812	1	0.0176715	_SCLK(su)EPYK_	SCLK(1)EPYK	33	-0.76518	60.436
Contig41858	1	0.0205612	_LVAVGYGIK(su)K_	LVAVGYGIK(1)K	183	-0.063805	53.756
Contig42134	1	0.000770501	_HYK(su)NAFHALSR_	HYK(1)NAFHALSR	148	-0.48928	73.435
Contig42338	1	0.00276728	_AILDK(su)SEQK_	AILDK(1)SEQK	256	0.76743	84.615
Contig42338	1	0.00116262	_DK(su)DLAHIESHMK_	DK(1)DLAHIESHMK	157	0.62472	66.538
Contig42338	0.999997	0.00346475	_DKDLAHIESHMK(su)NFK_	DKDLAHIESHMK(1)NFK	167	0.20334	66.674
Contig42338	1	0.0025081	_GK(su)ECIPFLEK_	GK(1)ECIPFLEK	98	-0.21289	69.721
Contig42338	1	0.0000265	_K(su)TALYDFHVENGGK_	K(1)TALYDFHVENGGK	41	0.45363	74.848
Contig42338	1	0.0127045	_TIGEITSGGFSPCLK(su)K_	TIGEITSGGFSPCLK(1)K	365	-1.9242	49.188
Contig42340	1	0.0129835	_EEDLK(su)GFR_	EEDLK(1)GFR	138	-0.28354	66.073
Contig42340	1	0.0000398	_HIEK(su)GAAVEAK_	HIEK(1)GAAVEAK	351	0.24434	116.03
Contig42340	1	0.0324967	_TLISGK(su)FPEGWEK_	TLISGK(1)FPEGWEK	384	1.4637	43.297
Contig42616	1	0.000200761	_AVSLVLPQLK(su)GK_	AVSLVLPQLK(1)GK	314	1.4019	77.062
Contig42616	1	0.0112161	_VIK(su)VVSNR_	VIK(1)VVSNR	162	1.2744	69.812
Contig42964	1	0.0000868	_NFNIIQK(su)QLGR_	NFNIIQK(1)QLGR	70	1.2548	94.692
Contig4298	1	0.00830149	_LSDYIGK(su)K_	LSDYIGK(1)K	115	0.13265	66.351
Contig43437	1	0.0011256	_LK(su)EYSINTK_	LK(1)EYSINTK	56	1.4075	96.342
Contig43475	1	0.00361791	_AQDYYK(su)PGSMSK_	AQDYYK(1)PGSMSK	186	0.28538	61.11
Contig4446	1	0.00219174	_GGVHIVK(su)TEK_	GGVHIVK(1)TEK	97	-0.50174	75.387

Contig4446	1	0.00104354	_TEK(su)AEDIAGK_	TEK(1)AEDIAGK	100	0.13169	83.862
Contig4446	1	0.0222781	_K(su)GGTSIEDLAEK_	K(1)GGTSIEDLAEK	160	-0.0070363	49.243
Contig4446	1	0.00276727	_PVNK(su)VYLCQK_	PVNK(1)VYLCQK	127	0.96362	67.414
Contig4446	1	0.0035222	_QK(su)EIFALR_	QK(1)EIFALR	259	0.84656	86.794
Contig4667	1	0.00478836	_EVLDIFK(su)AK_	EVLDIFK(1)AK	219	0.64348	74.464
Contig4938	1	0.0296884	_ANSLTDEDIK(su)ALTK_	ANSLTDEDIK(1)ALTK	229	0.1694	46.68
Contig6045	1	0.032895	_GSDWIVNEM(ox)K(su)K_	GSDWIVNEMK(1)K	101	0.5783	40.015
Contig6724	1	0.0044231	_MAFK(su)STR_	MAFK(1)STR	130	-1.0633	91.626
Contig7357	1	0.0173297	_TVPK(su)TVENFR_	TVPK(1)TVENFR	56	-0.29476	55.314
Contig7499	1	0.00163727	_AVLIDK(su)DQNPK_	AVLIDK(1)DQNPK	348	0.57976	69.721
Contig7684	1	0.000886866	_FTTFK(su)PLGDR_	FTTFK(1)PLGDR	61	-0.55004	83.499
Contig7684	1	0.00182109	_VLIK(su)VQEAEEK_	VLIK(1)VQEAEEK	168	2.6826	67.385
Contig7916	1	0.000075	_AAQLGLK(su)TTCIEK_	AAQLGLK(1)TTCIEK	81	-1.9811	79.875
Contig7916	1	0.00766146	_TTCIEK(su)R_	TTCIEK(1)R	87	0.075764	82.671
Contig7916	1	0.0029891	_GIEGLFK(su)K_	GIEGLFK(1)K	157	0.23938	75.109
Contig7916	1	0.0000311	_GLGLEELGVK(su)LDK_	GLGLEELGVK(1)LDK	342	0.04487	88.101
Contig7916	1	0.00732566	_IIAEK(su)ETDK_	IIAEK(1)ETDK	457	0.16151	68.657
Contig7916	1	0.00479855	_SFQK(su)TLEK_	SFQK(1)TLEK	276	0.32417	83.862
Contig7916	1	0.00291451	_MK(su)AVTGLTK_	MK(1)AVTGLTK	143	-1.3449	85.533
Contig8228	1	0.00397711	_NAELDPEK(su)R_	NAELDPEK(1)R	219	0.13007	79.148
Contig8619	1	0.0031127	_AVIAK(su)SFER_	AVIAK(1)SFER	803	-0.49474	82.645
Contig8619	1	0.0292188	_LLNGEVGPK(su)TIHIPTGEK_	LLNGEVGPK(1)TIHIPTGEK	745	-0.012437	49.537
Contig8619	1	0.00169554	_NCDNFQVTK(su)ADVEK_	NCDNFQVTK(1)ADVEK	61	0.59898	60.55
Contig8619	1	0.00279929	_YLLK(su)SGLQK_	YLLK(1)SGLQK	488	0.68523	83.948
Contig8749	1	0.0358827	_GQK(su)EYLR_	GQK(1)EYLR	222	0.11459	56.091
Contig8863	1	0.0017242	_HVLK(su)SEGGIR_	HVLK(1)SEGGIR	178	0.043877	77.662
Contig8976	1	0.0235044	_IYENTK(su)TIK_	IYENTK(1)TIK	159	0.53429	53.751
Contig9227	1	2E-19	_ANIK(su)AIQEASESGVK_	ANIK(1)AIQEASESGVK	148	0.66851	145.43
Contig9268	1	0.00540166	_GK(su)IPASEFR_	GK(1)IPASEFR	121	0.082543	73.233
Contig9515	1	0.00107676	_ASCISFK(su)DSACR_	ASCISFK(1)DSACR	81	-2.0227	67.252

Table S3. The number of succinylated sites in each succinylated protein.

Protein	number of sites
Contig9786	1
Contig9515	1
Contig9268	1
Contig9227	1
Contig8976	1
Contig8863	1
Contig8749	1
Contig8619	4
Contig8228	1
Contig8119	1
Contig7916	7
Contig7684	2
Contig7499	1
Contig7396	1
Contig7357	1
Contig6895	2
Contig6724	1
Contig6210	1
Contig6045	1
Contig5689	2
Contig5591	1
Contig4938	1
Contig4667	1
Contig454	1
Contig4446	5
Contig43475	1
Contig43437	1
Contig43032	1
Contig4298	1
Contig42964	1
Contig42616	2
Contig42340	3
Contig42338	6
Contig42134	1
Contig41858	1
Contig41812	1
Contig41772	4
Contig4152	1
Contig40889	2
Contig39434	2
Contig39307	1
Contig392	3
Contig38987	3

Contig38899	3
Contig38810	1
Contig38639	1
Contig37760	1
Contig3710	3
Contig36589	1
Contig36475	2
Contig36350	1
Contig35931	1
Contig35859	5
Contig3575	1
Contig35702	1
Contig35544	1
Contig35316	1
Contig35287	1
Contig35181	1
Contig35075	1
Contig34873	2
Contig34759	3
Contig34715	1
Contig34627	2
Contig34617	1
Contig3457	1
Contig34414	1
Contig33154	3
Contig33055	7
Contig32731	3
Contig3264	1
Contig32590	1
Contig32572	3
Contig32510	3
Contig32342	1
Contig32159	1
Contig3195	1
Contig31735	2
Contig31013	3
Contig30828	1
Contig3071	2
Contig3062	1
Contig30441	1
Contig30157	1
Contig29956	5
Contig29951	1
Contig29422	1
Contig29215	2
Contig28856	1

Contig28454	1
Contig28177	1
Contig28157	2
Contig28049	1
Contig2798	2
Contig27826	1
Contig27743	1
Contig27558	1
Contig27136	3
Contig27115	2
Contig26908	1
Contig26853	1
Contig26788	2
Contig26568	1
Contig26567	1
Contig26084	1
Contig25940	1
Contig25868	1
Contig25847	2
Contig25672	2
Contig25601	1
Contig25574	1
Contig25407	4
Contig25285	1
Contig2510	1
Contig24949	1
Contig24602	2
Contig24557	1
Contig24498	1
Contig24406	1
Contig23978	1
Contig23929	1
Contig23896	1
Contig23293	1
Contig23168	2
Contig23147	1
Contig22710	1
Contig22504	1
Contig22424	1
Contig22408	1
Contig2229	1
Contig22170	1
Contig21943	1
Contig21854	1
Contig2176	2
Contig21709	1

Contig21609	1
Contig21422	1
Contig21350	1
Contig21171	3
Contig21087	3
Contig20924	3
Contig2087	2
Contig2068	1
Contig20543	1
Contig19753	1
Contig19663	1
Contig19607	1
Contig19420	7
Contig1890	1
Contig18880	1
Contig18557	1
Contig18385	4
Contig18104	1
Contig18033	6
Contig17946	1
Contig17880	1
Contig17831	1
Contig17332	2
Contig17296	3
Contig16920	1
Contig16895	1
Contig16825	2
Contig16402	1
Contig1604	1
Contig15920	1
Contig15874	1
Contig15732	2
Contig15684	2
Contig15674	6
Contig15471	3
Contig15015	1
Contig14654	1
Contig14456	3
Contig14376	1
Contig1425	3
Contig14144	2
Contig136	2
Contig13439	2
Contig13371	1
Contig13277	1
Contig13241	2

Contig13164	2
Contig1305	1
Contig12663	1
Contig12483	1
Contig12178	1
Contig12149	2
Contig11938	1
Contig11832	3
Contig11799	1
Contig10828	1
Contig10651	1
Contig10551	1

Table S4: Information of succinylated proteins involved in glycolysis, pyruvate metabolism and TCA cycle.

Protein	Enzyme Nomenclature	Abbreviation	Function	Sites
Contig8976	EC:5.4.2.2	PGM	Alpha-D-phosphohexomutase	1
Contig25672	EC:4.1.2.13	ALDO	fructose-bisphosphate aldolase	2
Contig32731	EC:2.7.2.3	PGK	phosphoglycerate kinase	3
Contig4152	EC:4.2.1.11	ENO	enolase	1
Contig32010	EC:1.2.4.1	PDHB	pyruvate dehydrogenase	1
Contig7916	EC:1.8.1.4	DLD	dihydrolipoamide dehydrogenase	7
Contig21171	EC:2.3.1.12	DLAT	pyruvate dehydrogenase	3
Contig32590	EC:1.2.1.3	ALDH	aldehyde dehydrogenase	1
Contig38639	EC:1.1.1.1	frmA	S-(hydroxymethyl)glutathione dehydrogenase	1
Contig18385	EC:2.3.3.1	CS	citrate synthase	4
Contig8619	EC:4.2.1.3	ACO	aconitate hydratase	4
Contig24602	EC:1.1.1.42	IDH1	isocitrate dehydrogenase	2
Contig26853	EC:1.1.1.41	IDH3	isocitrate dehydrogenase (NAD+)	1
Contig2087	EC:1.2.4.2	OGDH	2-oxoglutarate dehydrogenase	2
Contig3710	EC:2.3.1.61	DLST	2-oxoglutarate dehydrogenase	3
Contig4446	EC:6.2.1.5	LSC1	succinyl-CoA synthetase	5
Contig18557	EC:6.2.1.4	LSC2	succinyl-CoA synthetase	1
Contig43437	EC:1.3.5.1	SDHA	succinate dehydrogenase	1
Contig4938	EC:1.1.1.37	MDH2	malate dehydrogenase	1

Table S5: Information of succinylated proteins involved in carbon fixation.

Protein	Enzyme Nomenclature		Function	Sites
Contig25672	EC:4.1.2.13	ALDO	fructose-bisphosphate aldolase	2
Contig25407	EC:3.1.3.37	SBPase	sedoheptulose-bisphosphatase	4
Contig42340	EC:2.2.1.1	Transketolase	transketolase	3
Contig15471	EC:4.1.1.39	RBCS	ribulose-bisphosphate carboxylase	3
Contig32731	EC:2.7.2.3	PGK	phosphoglycerate kinase	3
Contig42616	EC:1.2.1.13	GAPA	glyceraldehyde-3-phosphate dehydrogenase	2
Contig25672	EC:4.1.2.13	ALDO	fructose-bisphosphate aldolase	2
Contig3195	EC:5.3.1.1	TPI	triosephosphate isomerase	1
Contig29422	EC:2.6.1.1	GOT2	aspartate aminotransferase	1
Contig4938	EC:1.1.1.37	MDH1	malate dehydrogenase	1
Contig16825	EC:1.1.1.39	MDH2	malate dehydrogenase	2
Contig34715	EC:2.6.1.2	GGAT	glutamate-glyoxylate aminotransferase	1

Table S6: The information of 162 succinylated proteins that involved in Protein-protein interactions (PPIs) network.

Node name	Protein	No. of m	Degree	MCODE_	Subcellular Locati
3694.grail3.0106005101	Contig41812	1	17	7.27	mitochondria
3694.gw1.X.1285.1	Contig3457	1	11	7.00	chloroplast
3694.estExt_Genewise1_v1.C_LG_XVII1357	Contig23929	1	26	7.27	mitochondria
3694.grail3.0010030801	Contig7916	7	26	6.98	chloroplast
3694.estExt_fgenesh4_pg.C_LG_I2224	Contig43437	1	23	8.18	mitochondria
3694.gw1.6399.1.1	Contig23168	2	15	7.47	plasma membrane
3694.estExt_fgenesh4_pg.C_LG_XVI0781	Contig21087	3	22	8.00	chloroplast
3694.estExt_fgenesh4_kg.C_LG_IX0036	Contig10551	1	22	8.18	mitochondria
3694.eugene3.00130573	Contig40889	2	15	8.00	cytosol
3694.estExt_fgenesh4_pm.C_LG_VIII0532	Contig39434	2	13	6.81	chloroplast
3694.estExt_fgenesh4_pm.C_LG_XVIII0241	Contig392	3	20	8.00	cytosol
3694.estExt_fgenesh4_pm.C_LG_VIII0144	Contig20924	3	16	8.18	cytosol
3694.estExt_fgenesh4_pg.C_LG_XIV0805	Contig2176	2	7	7.00	cytosol
3694.estExt_Genewise1_v1.C_LG_IV0774	Contig25672	2	13	6.81	chloroplast
3694.estExt_fgenesh4_pm.C_LG_IX0030	Contig26853	1	22	8.00	chloroplast
3694.eugene3.00021349	Contig42340	3	13	6.81	chloroplast
3694.eugene3.00040033	Contig25601	1	19	7.27	chloroplast
3694.eugene3.00180798	Contig27826	1	13	6.81	cytosol
3694.eugene3.00151093	Contig4152	1	8	6.81	cytosol
3694.estExt_Genewise1_v1.C_LG_VIII1306	Contig22170	1	20	7.27	chloroplast
3694.gw1.20770.4.1	Contig33055	7	15	7.47	cytosol
3694.estExt_fgenesh4_pg.C_LG_X1053	Contig14456	3	20	7.27	mitochondria
3694.gw1.XIV.3012.1	Contig29422	1	20	8.00	mitochondria
3694.estExt_fgenesh4_pm.C_LG_XII0223	Contig34873	2	19	7.27	mitochondria
3694.fgenesh4_pg.C_LG_X001588	Contig34627	2	21	8.00	chloroplast
3694.estExt_fgenesh4_pm.C_LG_X0655	Contig32731	3	16	6.81	chloroplast
3694.estExt_Genewise1_v1.C_LG_IX1362	Contig3195	1	12	6.81	chloroplast
3694.gw1.6399.6.1	Contig9268	1	15	7.47	cytosol
3694.estExt_fgenesh4_pm.C_LG_V0707	Contig42616	2	7	7.00	chloroplast
3694.gw1.82.189.1	Contig2068	1	24	6.53	chloroplast
3694.grail3.0001073802	Contig11832	3	19	6.00	cytosol
3694.gw1.XIV.1833.1	Contig32159	1	9	7.00	chloroplast
3694.grail3.0012036701	Contig43475	1	21	6.53	chloroplast
3694.estExt_Genewise1_v1.C_LG_XVI2794	Contig28856	1	26	5.80	chloroplast
3694.estExt_Genewise1_v1.C_LG_I8285	Contig11799	1	19	6.53	chloroplast
3694.estExt_fgenesh4_pg.C_LG_I1607	Contig18033	6	22	7.00	cytosol
3694.estExt_fgenesh4_pm.C_1480010	Contig18385	4	26	5.80	cytosol
3694.estExt_fgenesh4_pg.C_LG_VIII0227	Contig21422	1	15	6.00	chloroplast
3694.estExt_fgenesh4_pm.C_LG_XIII0026	Contig21350	1	19	6.53	chloroplast
3694.fgenesh4_pg.C_LG_III000191	Contig21171	3	16	6.00	mitochondria
3694.grail3.0023029001	Contig8228	1	10	7.00	chloroplast
3694.eugene3.00020518	Contig1305	1	14	6.53	cytosol
3694.estExt_fgenesh4_pg.C_660263	Contig26908	1	8	2.40	mitochondria
3694.estExt_Genewise1_v1.C_280199	Contig7684	2	6	2.20	chloroplast
3694.estExt_fgenesh4_pm.C_LG_I0780	Contig13164	2	6	2.20	chloroplast

3694.estExt_fgenesh4_pm.C_LG_I0166	Contig17296	3	8	2.40	mitochondria
3694.estExt_Genewise1_v1.C_LG_III2625	Contig17332	2	8	2.40	chloroplast
3694.gw1.XVII.594.1	Contig20543	1	8	2.00	chloroplast
3694.estExt_Genewise1_v1.C_LG_XI2343	Contig26568	1	8	2.00	nuclear
3694.estExt_fgenesh4_pg.C_LG_XIII0274	Contig25940	1	6	2.20	cytosol
3694.eugene3.00140509	Contig35181	1	5	4.00	cytosol
3694.gw1.683.11.1	Contig5591	1	7	4.00	cytosol
3694.eugene3.00050364	Contig28049	1	8	4.00	cytosol
3694.estExt_Genewise1_v1.C_LG_X3287	Contig19607	1	6	4.00	cytosol
3694.estExt_Genewise1_v1.C_LG_XI0817	Contig19753	1	7	4.00	cytosol
3694.estExt_Genewise1_v1.C_LG_XI2803	Contig3710	3	20	5.73	mitochondria
3694.grail3.0044016201	Contig24602	2	18	4.80	cytosol
3694.estExt_fgenesh4_pm.C_LG_I0194	Contig25285	1	15	4.67	chloroplast
3694.estExt_Genewise1_v1.C_LG_XIV0188	Contig4446	5	19	4.93	mitochondria
3694.estExt_fgenesh4_pg.C_LG_II2062	Contig8619	4	10	5.50	cytosol
3694.estExt_fgenesh4_pm.C_LG_V0127	Contig18557	1	15	4.93	mitochondria
3694.gw1.X.5451.1	Contig29215	2	16	4.67	mitochondria
3694.gw1.VII.3485.1	Contig13241	2	13	4.76	chloroplast
3694.eugene3.00150408	Contig27743	1	19	3.89	chloroplast
3694.gw1.III.1082.1	Contig15732	2	7	4.00	cytosol
3694.estExt_fgenesh4_pg.C_2520014	Contig42338	6	15	4.00	chloroplast
3694.estExt_fgenesh4_pm.C_LG_X0942	Contig5689	2	9	4.00	mitochondria
3694.estExt_fgenesh4_pm.C_280146	Contig30441	1	10	4.11	chloroplast
3694.gw1.VIII.591.1	Contig34617	1	5	3.00	cytoskeleton
3694.gw1.X.2081.1	Contig39307	1	6	3.00	chloroplast
3694.estExt_fgenesh4_pg.C_LG_XVII0246	Contig8749	1	3	3.00	cytosol
3694.estExt_fgenesh4_pg.C_LG_VIII0421	Contig2087	2	26	4.57	chloroplast,mitocho
3694.grail3.0008003701	Contig21609	1	18	2.12	cytosol
3694.eugene3.00060644	Contig31735	2	4	3.00	cytosol
3694.gw1.X.1500.1	Contig15015	1	9	4.00	chloroplast
3694.fgenesh4_pm.C_LG_XIV000150	Contig36350	1	1	0.00	chloroplast
3694.grail3.0010031101	Contig1604	1	4	3.00	chloroplast
3694.eugene3.00130195	Contig15874	1	6	4.00	plasma membrane
3694.eugene3.00010969	Contig6895	2	1	0.00	plasma membrane
3694.gw1.513.7.1	Contig1890	1	11	2.25	extracellular
3694.gw1.14458.2.1	Contig15674	6	11	1.58	mitochondria
3694.estExt_fgenesh4_pm.C_LG_IX0400	Contig29956	5	10	1.20	chloroplast
3694.estExt_fgenesh4_pm.C_LG_VI0678	Contig26084	1	16	3.33	chloroplast
3694.eugene3.00100819	Contig26788	2	16	4.32	chloroplast
3694.estExt_fgenesh4_pg.C_LG_X0989	Contig8976	1	14	6.00	cytosol
3694.grail3.0021033801	Contig38639	1	2	0.67	cytosol
3694.gw1.28.53.1	Contig19663	1	4	3.00	cytosol
3694.eugene3.00101874	Contig25407	4	9	4.00	chloroplast
3694.gw1.XVIII.3218.1	Contig25847	2	5	3.00	cytosol
3694.estExt_fgenesh4_pg.C_1500058	Contig12483	1	10	1.20	chloroplast
3694.estExt_Genewise1_v1.C_LG_XVI3692	Contig3575	1	4	1.40	extracellular

3694.fgenesh4_pm.C_LG_I000162	Contig12149	2	14	2.78	chloroplast
3694.estExt_fgenesh4_pg.C_LG_II1218	Contig16825	2	10	1.67	chloroplast
3694.eugene3.00021450	Contig34414	1	4	2.00	chloroplast,mitocho
3694.fgenesh4_pg.C_LG_IV000431	Contig4938	1	15	3.89	mitochondria
3694.estExt_fgenesh4_pm.C_LG_II0915	Contig32590	1	5	1.20	mitochondria
3694.estExt_fgenesh4_pm.C_290034	Contig14144	2	9	4.00	cytosol
3694.gw1.VII.539.1	Contig27136	3	2	0.67	chloroplast
3694.eugene3.00081764	Contig34715	1	12	5.00	cytosol
3694.estExt_Genewise1_v1.C_LG_II2125	Contig35316	1	4	1.67	cytosol
3694.estExt_fgenesh4_pm.C_LG_IV0266	Contig33154	3	4	1.67	cytosol
3694.eugene3.01460031	Contig38899	3	7	1.20	cytosol
3694.estExt_fgenesh4_kg.C_LG_IV0030	Contig12663	1	8	4.29	cytosol
3694.estExt_fgenesh4_pg.C_LG_II1351	Contig18880	1	2	0.67	chloroplast
3694.gw1.VII.3752.1	Contig8119	1	2	0.67	chloroplast
3694.estExt_fgenesh4_pg.C_LG_VII1183	Contig17880	1	6	3.00	nuclear
3694.estExt_fgenesh4_pg.C_LG_II1684	Contig1425	3	4	3.00	chloroplast
3694.eugene3.00010884	Contig31013	3	5	3.00	chloroplast
3694.estExt_fgenesh4_pg.C_LG_X0463	Contig6045	1	11	5.36	mitochondria
3694.eugene3.01230087	Contig15471	3	9	2.14	chloroplast
3694.eugene3.00110470	Contig2510	1	5	3.47	chloroplast
3694.estExt_fgenesh4_pm.C_LG_I0932	Contig24949	1	7	2.20	chloroplast
3694.estExt_fgenesh4_pg.C_1660047	Contig11938	1	3	3.00	chloroplast
3694.estExt_fgenesh4_pg.C_LG_I1460	Contig6724	1	5	3.47	mitochondria
3694.estExt_Genewise1_v1.C_LG_XIII3158	Contig32510	3	6	2.20	mitochondria
3694.fgenesh4_pm.C_scaffold_163000009	Contig13277	1	3	2.00	cytosol
3694.fgenesh4_pm.C_LG_I001114	Contig21854	1	10	1.20	mitochondria
3694.estExt_fgenesh4_pg.C_LG_X2033	Contig17946	1	1	0.00	chloroplast
3694.grail3.0202001702	Contig22710	1	4	4.00	chloroplast
3694.estExt_fgenesh4_pm.C_LG_V0695	Contig35287	1	11	1.71	peroxisome
3694.grail3.0002075501	Contig17831	1	5	2.70	nuclear
3694.eugene3.00160660	Contig4298	1	9	2.70	chloroplast
3694.grail3.0028013201	Contig28157	2	2	2.00	cytosol
3694.estExt_fgenesh4_pg.C_LG_X0791	Contig35702	1	3	0.50	nuclear
3694.estExt_fgenesh4_pg.C_LG_XIV1000	Contig41772	4	6	3.00	mitochondria
3694.estExt_Genewise1_v1.C_LG_I2532	Contig3264	1	4	2.00	chloroplast
3694.gw1.XVIII.2487.1	Contig25574	1	9	2.40	chloroplast
3694.gw1.130.154.1	Contig24406	1	9	4.29	chloroplast
3694.estExt_fgenesh4_pm.C_LG_VII0233	Contig35859	5	8	2.70	chloroplast
3694.gw1.VI.2058.1	Contig36475	2	19	4.12	chloroplast
3694.eugene3.00013054	Contig16402	2	3	0.50	nuclear
3694.eugene3.00091463	Contig41858	1	6	3.00	nuclear
3694.estExt_fgenesh4_pm.C_LG_XIV0571	Contig23896	1	3	0.50	chloroplast
3694.eugene3.00141466	Contig28454	1	3	1.67	cytosol
3694.eugene3.29900002	Contig32342	1	3	0.50	chloroplast
3694.estExt_fgenesh4_pg.C_LG_VI1023	Contig7499	1	3	1.67	chloroplast
3694.eugene3.00140133	Contig38987	3	1	0.00	chloroplast

3694.grail3.0118000701	Contig8863	1	5	2.00	chloroplast
3694.estExt_fgenesh4_pg.C_400150	Contig27558	1	6	2.70	chloroplast
3694.estExt_Genewise1_v1.C_LG_II2900	Contig6210	1	1	0.00	chloroplast
3694.gw1.II.2305.1	Contig14654	1	1	0.00	cytosol
3694.estExt_Genewise1_v1.C_LG_XI3916	Contig21709	1	1	0.00	chloroplast
3694.grail3.0024013201	Contig4667	1	3	2.00	chloroplast
3694.eugene3.00131316	Contig26567	1	2	0.67	chloroplast
3694.estExt_Genewise1_v1.C_LG_IX3737	Contig7357	1	1	0.00	cytosol
3694.gw1.127.120.1	Contig35075	1	3	1.67	nuclear
3694.eugene3.00020135	Contig32572	3	3	0.50	chloroplast
3694.eugene3.00031909	Contig454	1	1	0.00	nuclear
3694.eugene3.00060732	Contig29951	1	1	0.00	chloroplast
3694.estExt_fgenesh4_pm.C_LG_XIX0169	Contig23978	1	1	0.00	cytosol
3694.estExt_fgenesh4_pm.C_LG_II0162	Contig10828	1	3	2.00	cytosol
3694.estExt_fgenesh4_pg.C_LG_VI0717	Contig30828	1	1	0.00	chloroplast
3694.estExt_fgenesh4_pg.C_LG_IV1470	Contig34759	3	2	2.00	chloroplast
3694.estExt_fgenesh4_pg.C_LG_III1822	Contig42134	1	1	0.00	chloroplast
3694.estExt_Genewise1_v1.C_LG_I0264	Contig35544	1	1	0.00	chloroplast
3694.fgenesh4_pm.C_LG_X000986	Contig22408	1	1	0.00	chloroplast
3694.gw1.X.2350.1	Contig7396	1	1	0.00	cytosol
3694.fgenesh4_pm.C_scaffold_166000008	Contig14376	1	1	0.00	chloroplast
3694.estExt_fgenesh4_kg.C_LG_X0015	Contig136	2	1	0.00	cytosol
3694.gw1.148.177.1	Contig27115	2	1	0.00	cytosol
3694.fgenesh4_pg.C_scaffold_135000030	Contig23147	1	1	0.00	chloroplast
3694.grail3.0113000401	Contig35931	1	1	0.00	cytosol

Table S7: The list of the proteins that classed into cluster 1.

Node name	Protein	No. of moc	Degree	MCODE_	Subcellular Location
3694.grail3.0106005101	Contig41812	1	17	7.27	mitochondria
3694.gw1.X.1285.1	Contig3457	1	11	7.00	chloroplast
3694.estExt_Genewise1_v1.C_LG_XVII1357	Contig23929	1	26	7.27	mitochondria
3694.grail3.0010030801	Contig7916	7	26	6.98	chloroplast
3694.estExt_fgenesh4_pg.C_LG_I2224	Contig43437	1	23	8.18	mitochondria
3694.gw1.6399.1.1	Contig23168	2	15	7.47	plasma membrane
3694.estExt_fgenesh4_pg.C_LG_XVI0781	Contig21087	3	22	8.00	chloroplast
3694.estExt_fgenesh4_kg.C_LG_IX0036	Contig10551	1	22	8.18	mitochondria
3694.eugene3.00130573	Contig40889	2	15	8.00	cytosol
3694.estExt_fgenesh4_pm.C_LG_VIII0532	Contig39434	2	13	6.81	chloroplast
3694.estExt_fgenesh4_pm.C_LG_XVIII0241	Contig392	3	20	8.00	cytosol
3694.estExt_fgenesh4_pm.C_LG_VIII0144	Contig20924	3	16	8.18	cytosol
3694.estExt_fgenesh4_pg.C_LG_XIV0805	Contig2176	2	7	7.00	cytosol
3694.estExt_Genewise1_v1.C_LG_IV0774	Contig25672	2	13	6.81	chloroplast
3694.estExt_fgenesh4_pm.C_LG_IX0030	Contig26853	1	22	8.00	chloroplast
3694.eugene3.00021349	Contig42340	3	13	6.81	chloroplast
3694.eugene3.00040033	Contig25601	1	19	7.27	chloroplast
3694.eugene3.00180798	Contig27826	1	13	6.81	cytosol
3694.eugene3.00151093	Contig4152	1	8	6.81	cytosol
3694.estExt_Genewise1_v1.C_LG_VIII1306	Contig22170	1	20	7.27	chloroplast
3694.gw1.20770.4.1	Contig33055	7	15	7.47	cytosol
3694.estExt_fgenesh4_pg.C_LG_X1053	Contig14456	3	20	7.27	mitochondria
3694.gw1.XIV.3012.1	Contig29422	1	20	8.00	mitochondria
3694.estExt_fgenesh4_pm.C_LG_XII0223	Contig34873	2	19	7.27	mitochondria
3694.fgenesh4_pg.C_LG_X001588	Contig34627	2	21	8.00	chloroplast
3694.estExt_fgenesh4_pm.C_LG_X0655	Contig32731	3	16	6.81	chloroplast
3694.estExt_Genewise1_v1.C_LG_IX1362	Contig3195	1	12	6.81	chloroplast
3694.gw1.6399.6.1	Contig9268	1	15	7.47	cytosol
3694.estExt_fgenesh4_pm.C_LG_V0707	Contig42616	2	7	7.00	chloroplast

Table S8: The list of the proteins that classed into cluster 2.

Node name	Protein	No. of mod	Degree	MCODE_S	Subcellular Location
3694.gw1.82.189.1	Contig2068	1	24	6.5333333	chloroplast
3694.grail3.0001073802	Contig11832	3	19	6	cytosol
3694.gw1.XIV.1833.1	Contig32159	1	9	7	chloroplast
3694.grail3.0012036701	Contig43475	1	21	6.5333333	chloroplast
3694.estExt_Genewise1_v1.C_LG_XVI2794	Contig28856	1	26	5.8	chloroplast
3694.estExt_Genewise1_v1.C_LG_I8285	Contig11799	1	19	6.5333333	chloroplast
3694.estExt_fgenesh4_pg.C_LG_I1607	Contig18033	6	22	7	cytosol
3694.estExt_fgenesh4_pm.C_1480010	Contig18385	4	26	5.8	cytosol
3694.estExt_fgenesh4_pg.C_LG_VIII0227	Contig21422	1	15	6	chloroplast
3694.estExt_fgenesh4_pm.C_LG_XIII0026	Contig21350	1	19	6.5333333	chloroplast
3694.fgenesh4_pg.C_LG_III000191	Contig21171	3	16	6	mitochondria
3694.grail3.0023029001	Contig8228	1	10	7	chloroplast
3694.eugene3.00020518	Contig1305	1	14	6.5333333	cytosol

Table S9: The list of the proteins that classed into cluster 3.

**Node name**	**Protein**	**No. of mod**	**Degree**	**MCODE_Score**	**Subcellular Location**
3694.estExt_fgenesh4_pg.C_660263	Contig26908	1	8	2.4	mitochondria
3694.estExt_Genewise1_v1.C_280199	Contig7684	2	6	2.2	chloroplast
3694.estExt_fgenesh4_pm.C_LG_I0780	Contig13164	2	6	2.2	chloroplast
3694.estExt_fgenesh4_pm.C_LG_I0166	Contig17296	3	8	2.4	mitochondria
3694.estExt_Genewise1_v1.C_LG_III2625	Contig17332	2	8	2.4	chloroplast
3694.gw1.XVII.594.1	Contig20543	1	8	2	chloroplast
3694.estExt_Genewise1_v1.C_LG_XI2343	Contig26568	1	8	2	nuclear
3694.estExt_fgenesh4_pg.C_LG_XIII0274	Contig25940	1	6	2.2	cytosol

Table S10: The list of the proteins that classed into cluster 4.

Node name	Protein	No. of sites	Degree	MCODE	Subcellular Location
3694.eugene3.00140509	Contig35181	1	5	4	cytosol
3694.gw1.683.11.1	Contig5591	1	7	4	cytosol
3694.eugene3.00050364	Contig28049	1	8	4	cytosol
3694.estExt_Genewise1_v1.C_LG_X3287	Contig19607	1	6	4	cytosol
3694.estExt_Genewise1_v1.C_LG_XI0817	Contig19753	1	7	4	cytosol

Table S11: The list of the proteins that classed into cluster 5.

Node name	Protein	No. of sites	Degree	MCODE	Subcellular Location
3694.estExt_Genewise1_v1.C_LG_XI2803	Contig3710	3	20	5.72727	mitochondria
3694.grail3.0044016201	Contig24602	2	18	4.8	cytosol
3694.estExt_fgenesh4_pm.C_LG_I0194	Contig25285	1	15	4.66667	chloroplast
3694.estExt_Genewise1_v1.C_LG_XIV0188	Contig4446	5	19	4.93333	mitochondria
3694.estExt_fgenesh4_pg.C_LG_II2062	Contig8619	4	10	5.5	cytosol
3694.estExt_fgenesh4_pm.C_LG_V0127	Contig18557	1	15	4.93333	mitochondria
3694.gw1.X.5451.1	Contig29215	2	16	4.66667	mitochondria
3694.gw1.VII.3485.1	Contig13241	2	13	4.7619	chloroplast

Table S12: Comparison of Taxus × media succinylated proteins with different reported species.

organism	no. of succinylated proteins	no. of succinylated sites	sites per protein
Taxus × media	193	325	1.68
Mycobacterium tuberculosis	686	1732	2.52
E.Coli	990	2572	2.6
Toxoplasma gondii	147	425	2.89
S. cerevisiae	474	1345	2.84
